# Supplementary material for: Extracting a low-dimensional description of multiple gene expression datasets reveals a potential driver for tumor-associated stroma in ovarian cancer
Source: Genome Med. 2016 Jun 10;8:66. doi: 10.1186/s13073-016-0319-7 (PMC4902951; doi:10.1186/s13073-016-0319-7)
Supplement: Additional file 1: Table S1. — The nine ovarian cancer gene expression datasets we used in the third set of experiments (biological application). (DOC 32 kb) [file 13073_2016_319_MOESM1_ESM.doc]

**Table S1 The nine ovarian cancer gene expression datasets we used in the third set of experiments (biological application).**

| **Name** | **Samples (n)** | **Genes (n)** | **Platform** |
| --- | --- | --- | --- |
| TCGA OV | 560 | 11,847 | Affymetrix HGU133A + Agilent g4502  + Human Exon array |
| GSE14764 | 80 | 11,942 | Affymetrix HGU133A |
| GSE26712 | 185 | 11,942 | Affymetrix HGU133A |
| GSE6008 | 99 | 11,942 | Affymetrix HGU133A |
| GSE18520 | 53 | 18,113 | Affymetrix HGU133Plus2 |
| GSE19829.GPL570 | 28 | 18,113 | Affymetrix HGU133Plus2 |
| GSE20565 | 140 | 18,113 | Affymetrix HGU133Plus2 |
| GSE30161 | 58 | 18,113 | Affymetrix HGU133Plus2 |
| GSE9899 | 295 | 18,113 | Affymetrix HGU133Plus2 |
