# Supplementary figures and images for: Extracting a low-dimensional description of multiple gene expression datasets reveals a potential driver for tumor-associated stroma in ovarian cancer
Source: Genome Med. 2016 Jun 10;8:66. doi: 10.1186/s13073-016-0319-7 (PMC4902951; doi:10.1186/s13073-016-0319-7)

Figure S1A

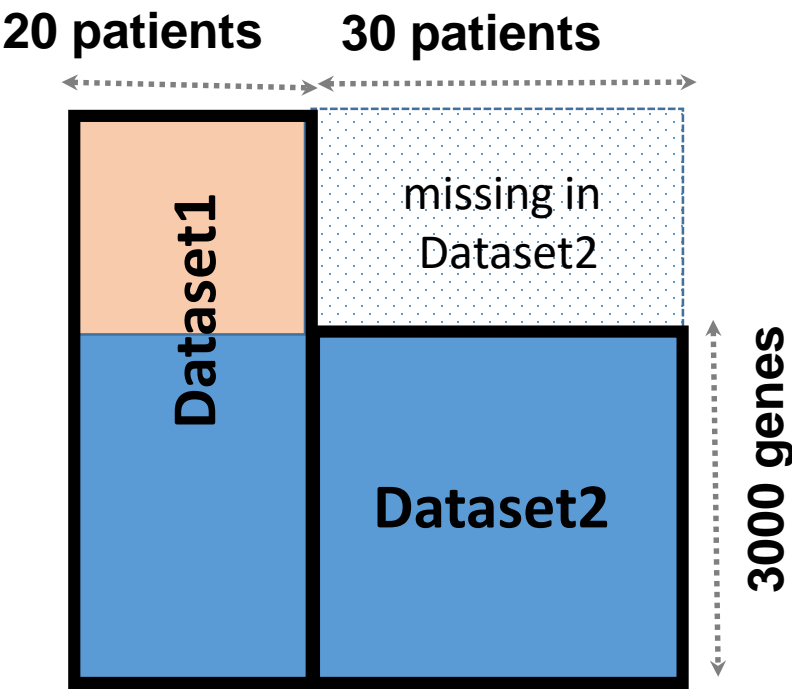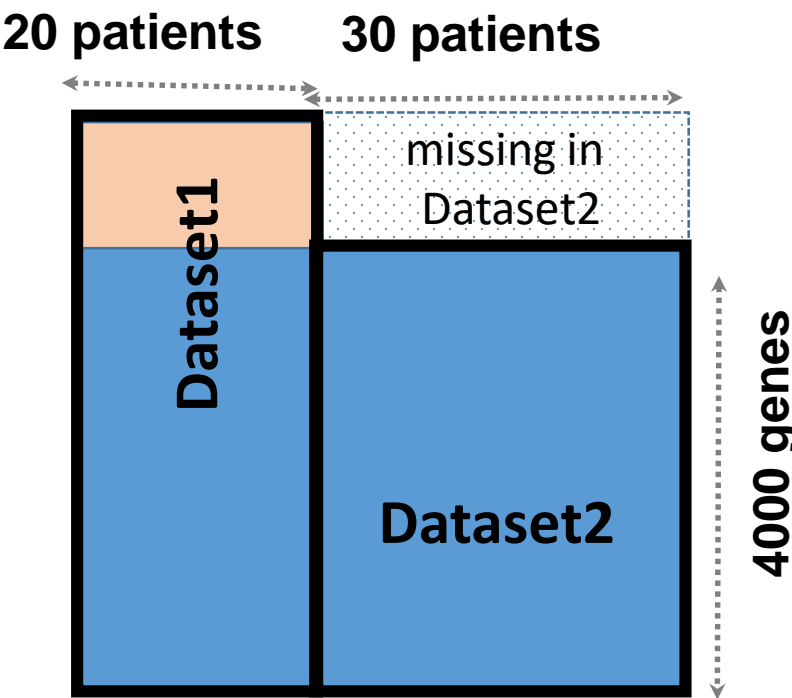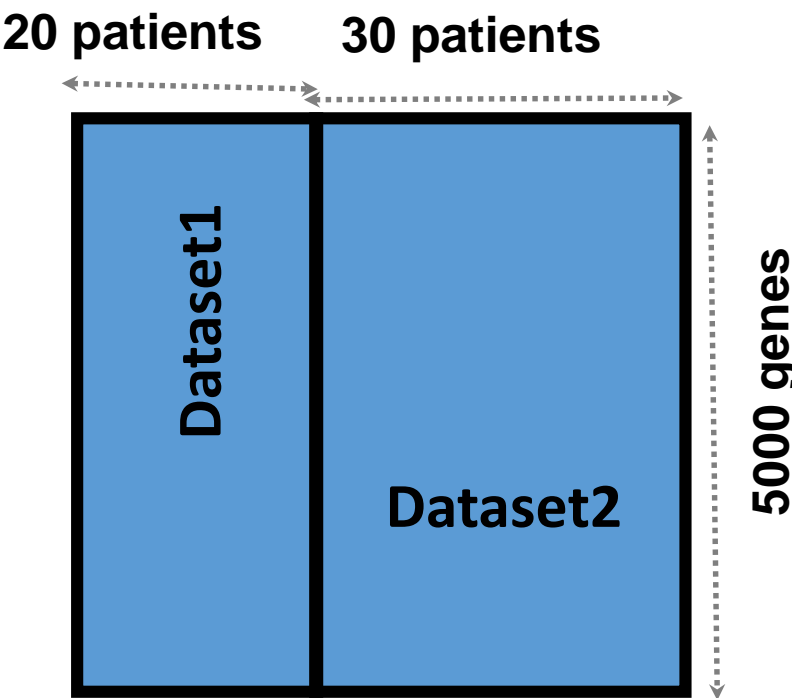

Figure S1B

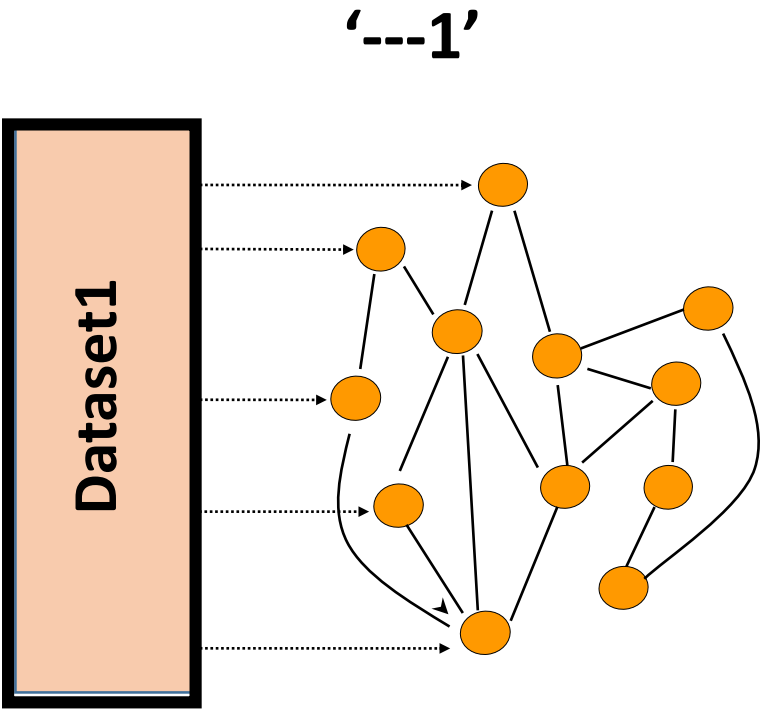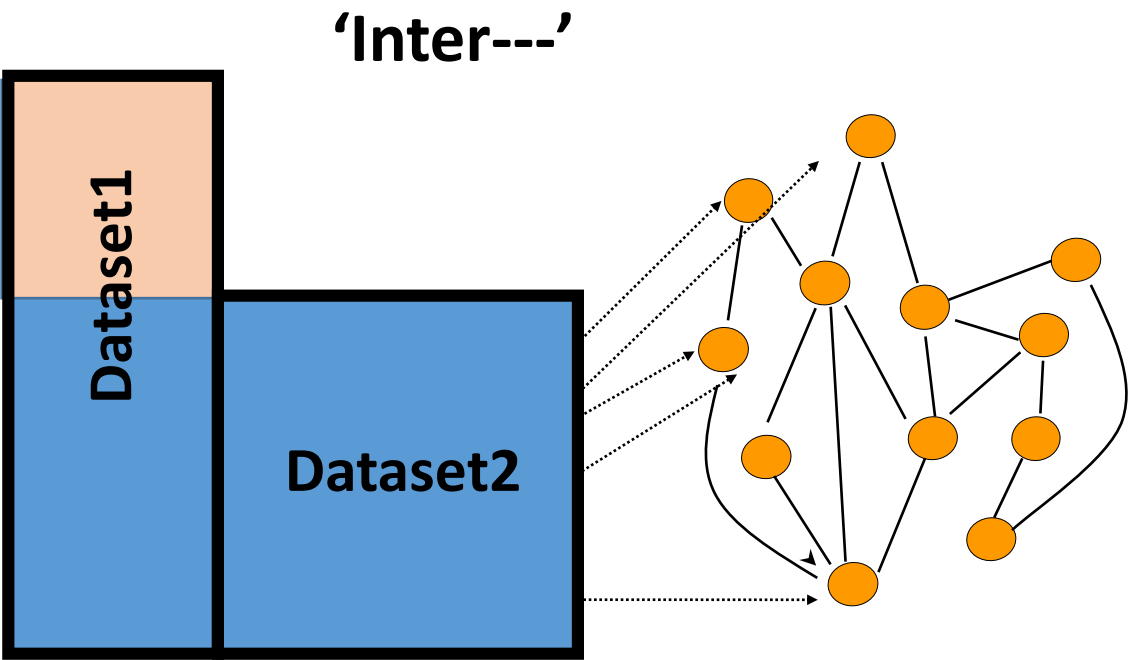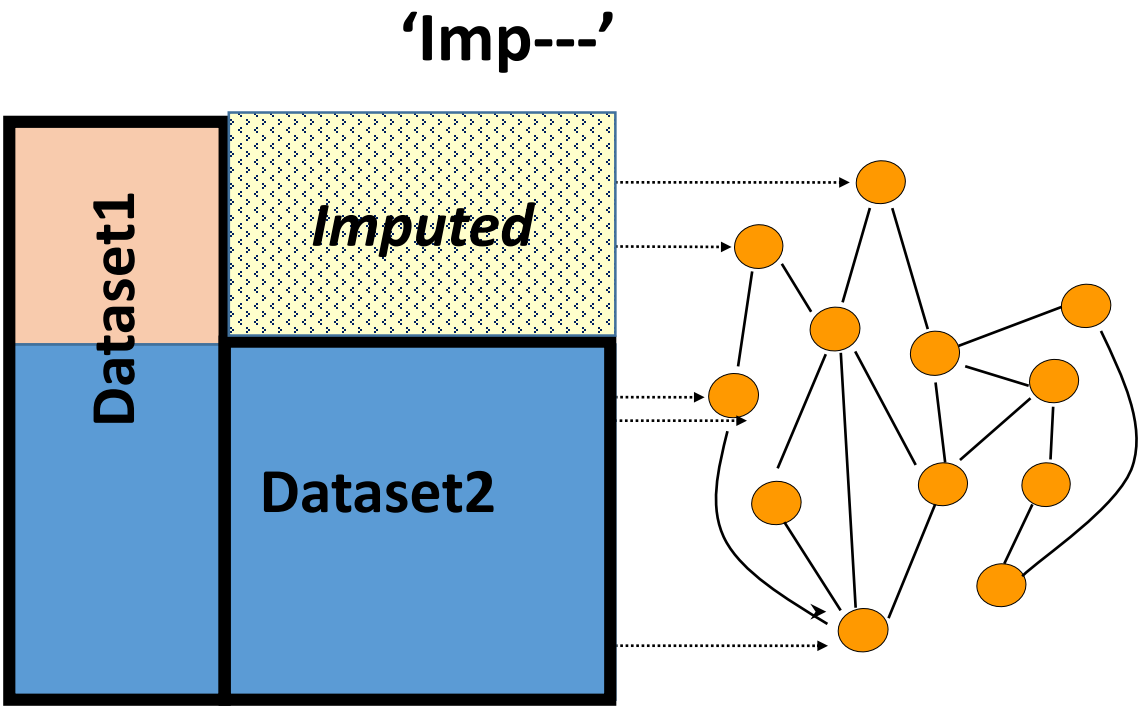

Supplement: Additional file 2: Figure S1. — A The synthetic data for the three generated simulation settings are illustrated. Rows represent genes and columns represent samples. In each setting, different amount of genes overlap between datasets (60 %, 80 %, and 100 %, respectively, from top to bottom). B Adapted learning ways for the alternative methods as explained in Table 1 are illustrated (“---1,” “Inter---,” and “Imp---,” respectively, from top to bottom). The first illustration corresponds to “--1” which performs standard learning from a single dataset. The second illustration corresponds to “Inter--” which learns the features using the overlapping genes and map data-specific genes to the learned features. The third illustration corresponds to “Imp--” which imputes the missing values and learns the features using the imputed data matrix. (PDF 292 kb) [file 13073_2016_319_MOESM2_ESM.pdf]

Figure S2A

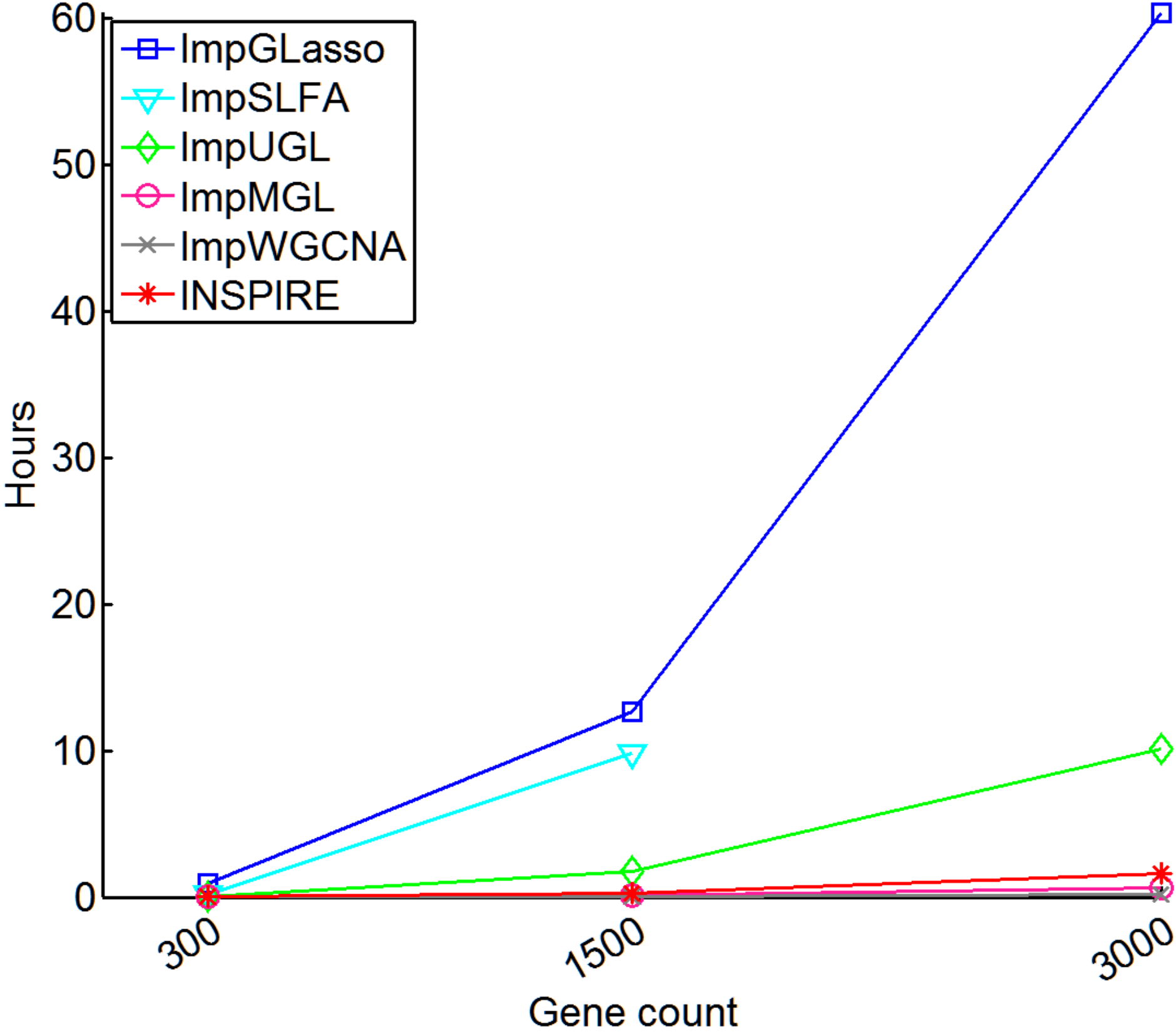

Figure S2B

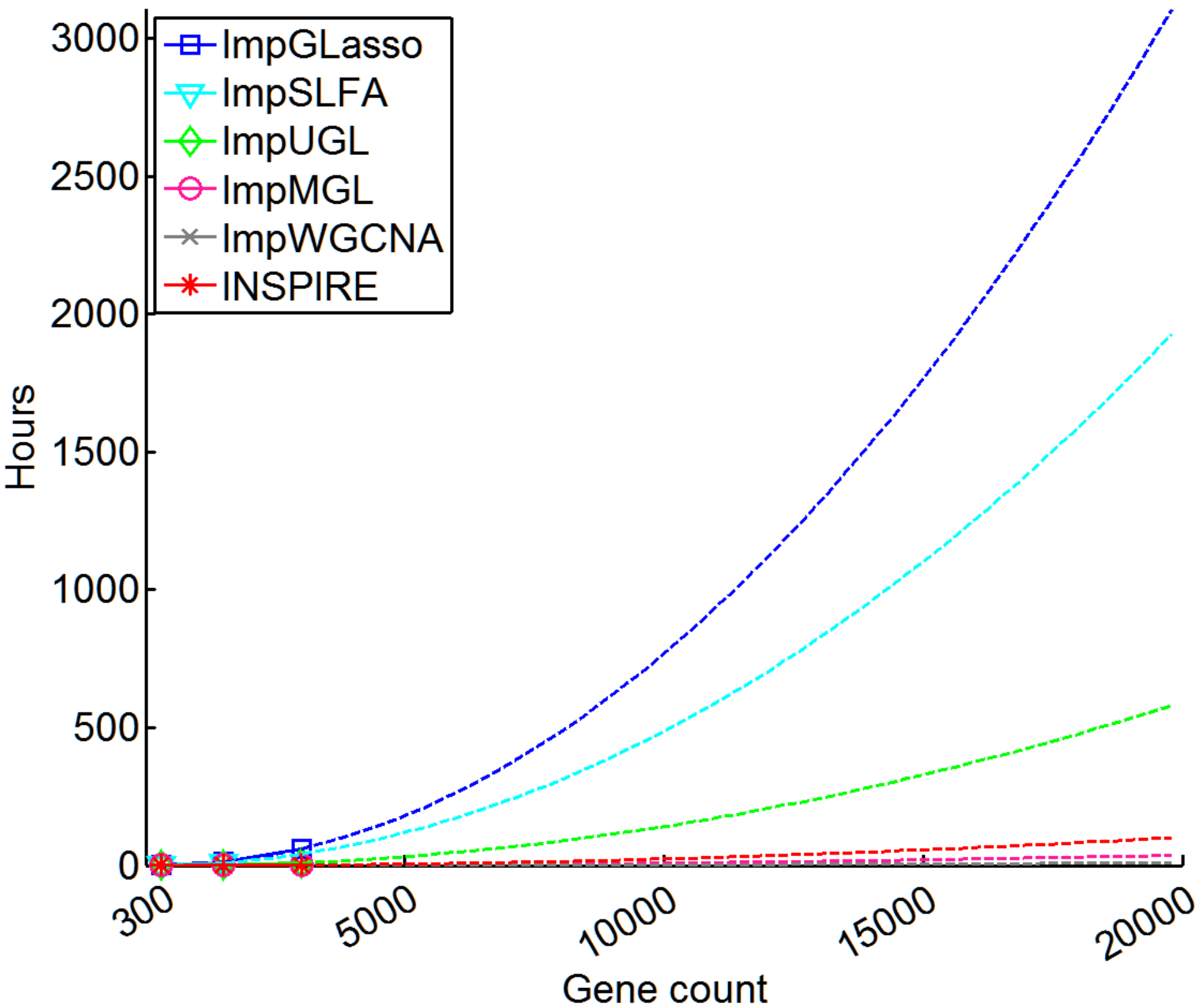

Supplement: Additional file 3: Figure S2. — A Runtimes (in hours on the y-axis) of INSPIRE and five state-of-the-art methods that learn a network of modules or genes from a single dataset is compared for varying gene counts (p = 300, p = 1500, and p = 3000 as shown on the x-axis) where the imputed data are used for the methods that are unable make use of multiple datasets (all except INSPIRE). p = 3000 cannot be shown for ImpSLFA since it is unable to run for the cases where the module count (k) exceeds the sample size. B The trend lines from a quadratic fit are added to show the estimated runtimes (in hours on the y-axis) for bigger p and genome-wide data (on the x-axis). (PDF 50 kb) [file 13073_2016_319_MOESM3_ESM.pdf]

Figure S3

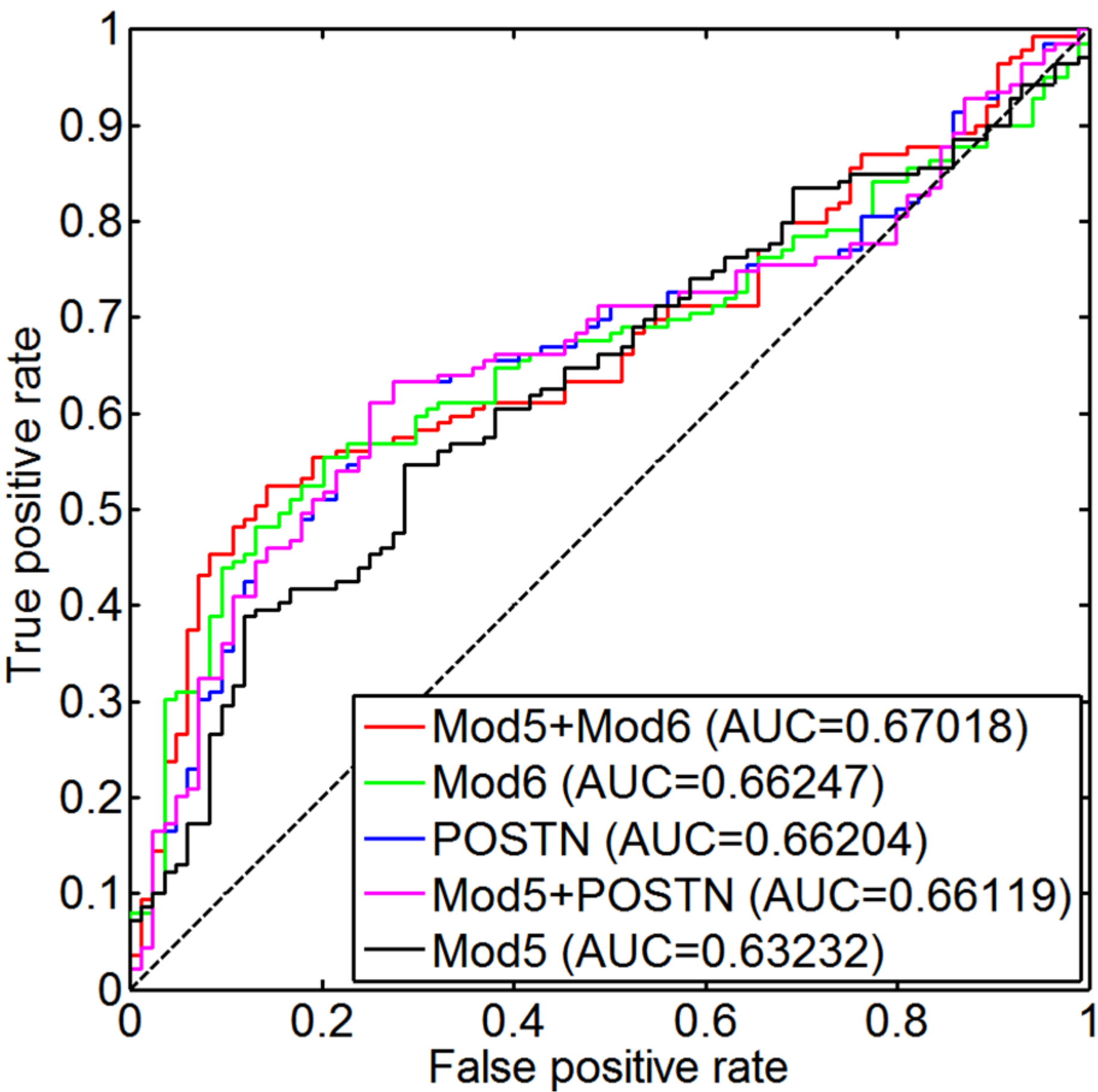

Supplement: Additional file 4: Figure S3. — ROC curve of the supervised models for resectability prediction trained in TCGA and tested in Tothill data. Different combinations of POSTN and the INSPIRE features corresponding to modules 5 and 6 are used for training each model. The clinical covariates age and stage are not included in the models. AUC of each model is shown in the legend. (PDF 139 kb) [file 13073_2016_319_MOESM4_ESM.pdf]

Figure S4A

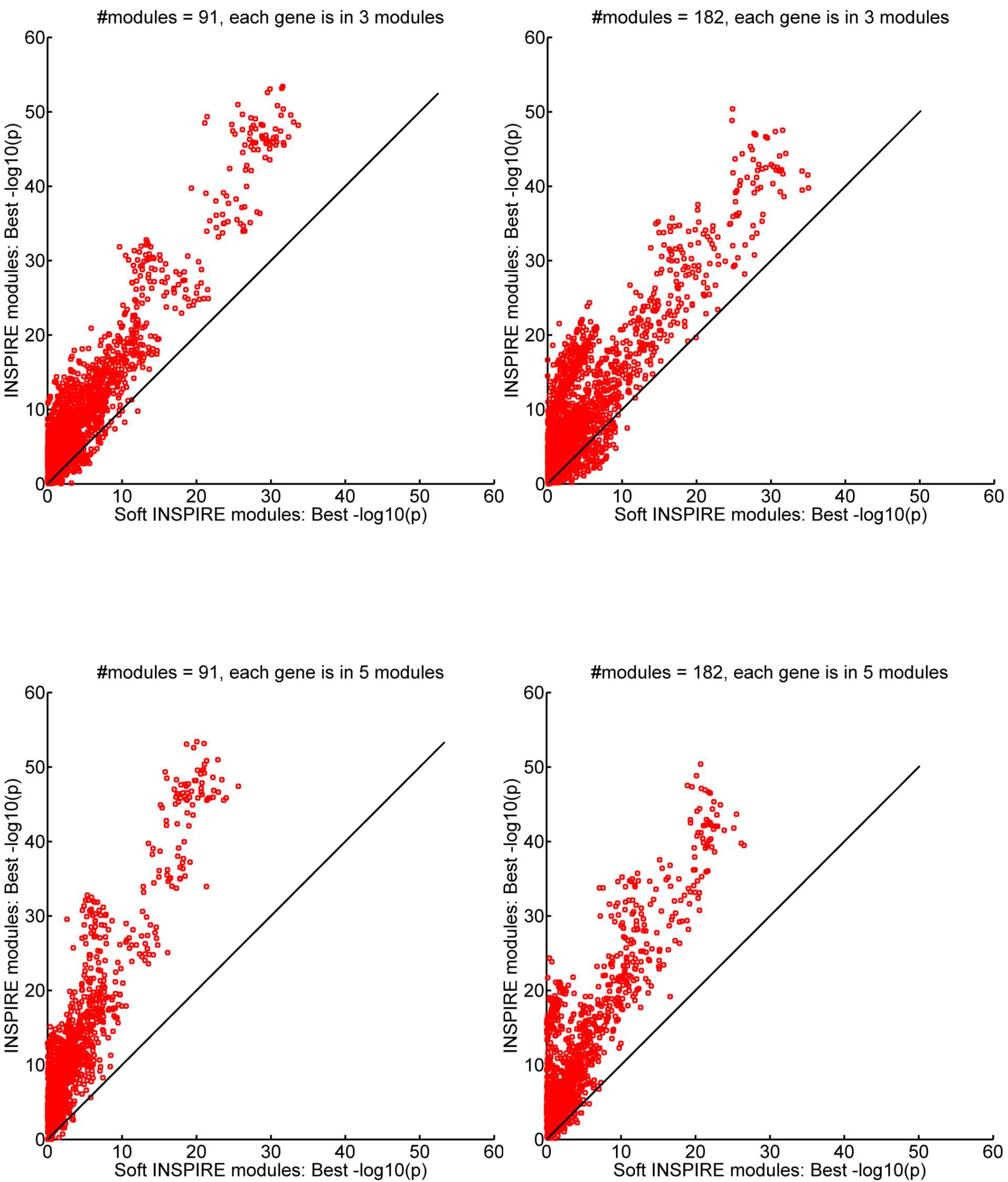

Figure S4B

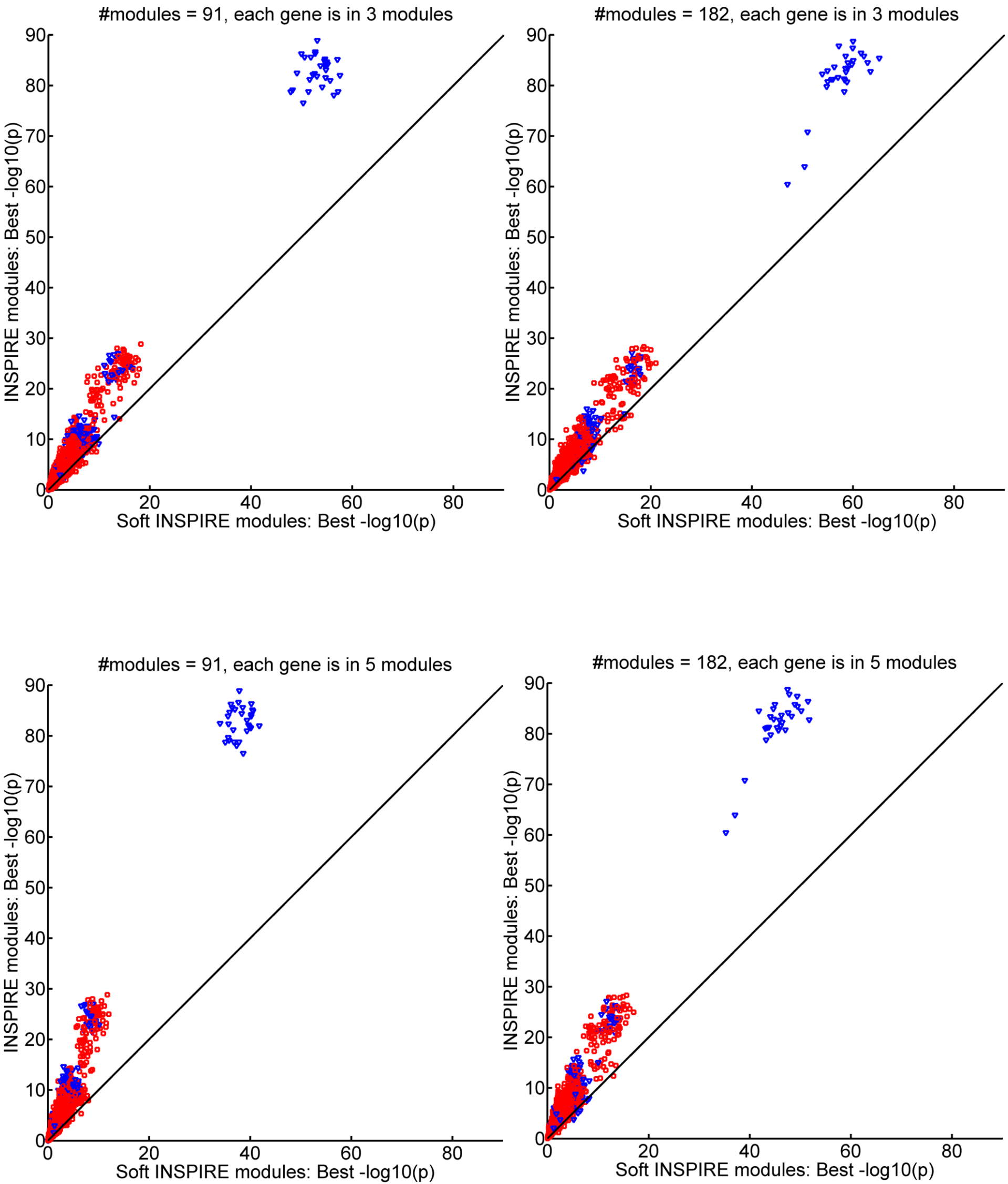

Supplement: Additional file 5: Figure S4. — A For k = 91 (left) and k = 182 (right), the best − log10 p from the functional enrichment of the modules learned by an INSPIRE extension that assigns each gene to more than one modules (on the x-axis) are compared to the best − log10 p from the functional enrichment of the modules learned by the proposed INSPIRE approach (on the y-axis). Each dot corresponds to a KEGG, Reactome, or BioCarta GeneSet and only the GeneSets with a Bonferroni corrected Fisher’s exact test p <0.05 in at least one of the compared two methods are shown on each plot. Two different versions of the INSPIRE extension was used in comparison; the one which assigns each gene to top three modules with highest potential to contain that gene (top) and the one which assigns each gene to top five modules (bottom). For both INSPIRE and alternative approach, the results from multiple runs are shown on each plot. B For k = 91 (left) and k = 182 (right), the best − log10 p from the ChEA enrichment of the modules learned by the INSPIRE extension (on the x-axis) are compared to the best − log10 p from the ChEA enrichment of the modules learned by INSPIRE (on the y-axis). Each dot is for a group of genes composed of a TF and its targets, and only the TFs with a Bonferroni corrected Fisher’s exact test p <0.05 in at least one of the compared two methods are shown on each plot. Two different versions of the INSPIRE extension was used in comparison; the one which assigns each gene to top three modules with highest potential to contain that gene (top) and the one which assigns each gene to top five modules (bottom). For both INSPIRE and alternative approach, the results from multiple runs are shown on each plot. (PDF 467 kb) [file 13073_2016_319_MOESM5_ESM.pdf]

**Figure S5A**

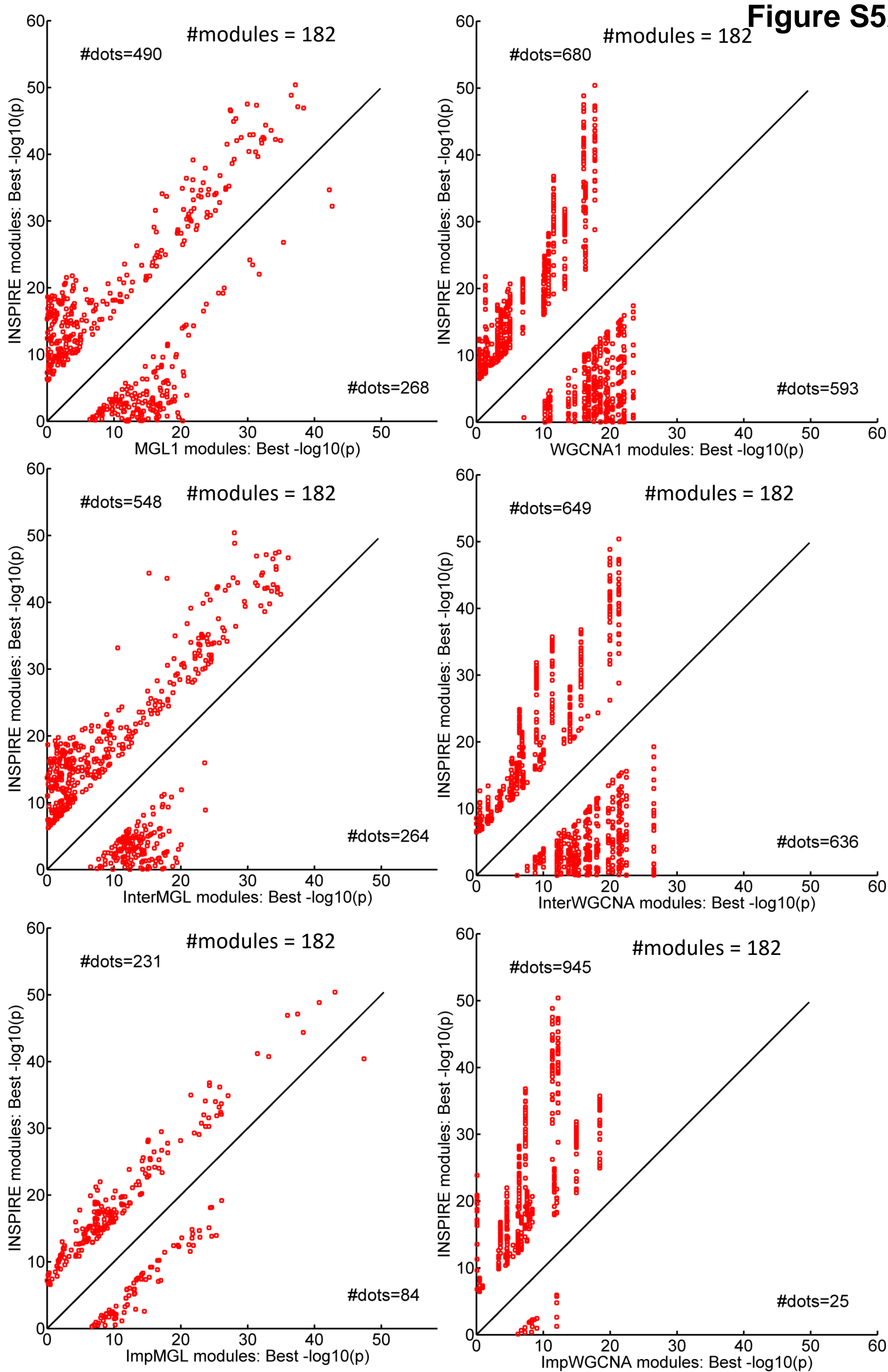

Figure S5B

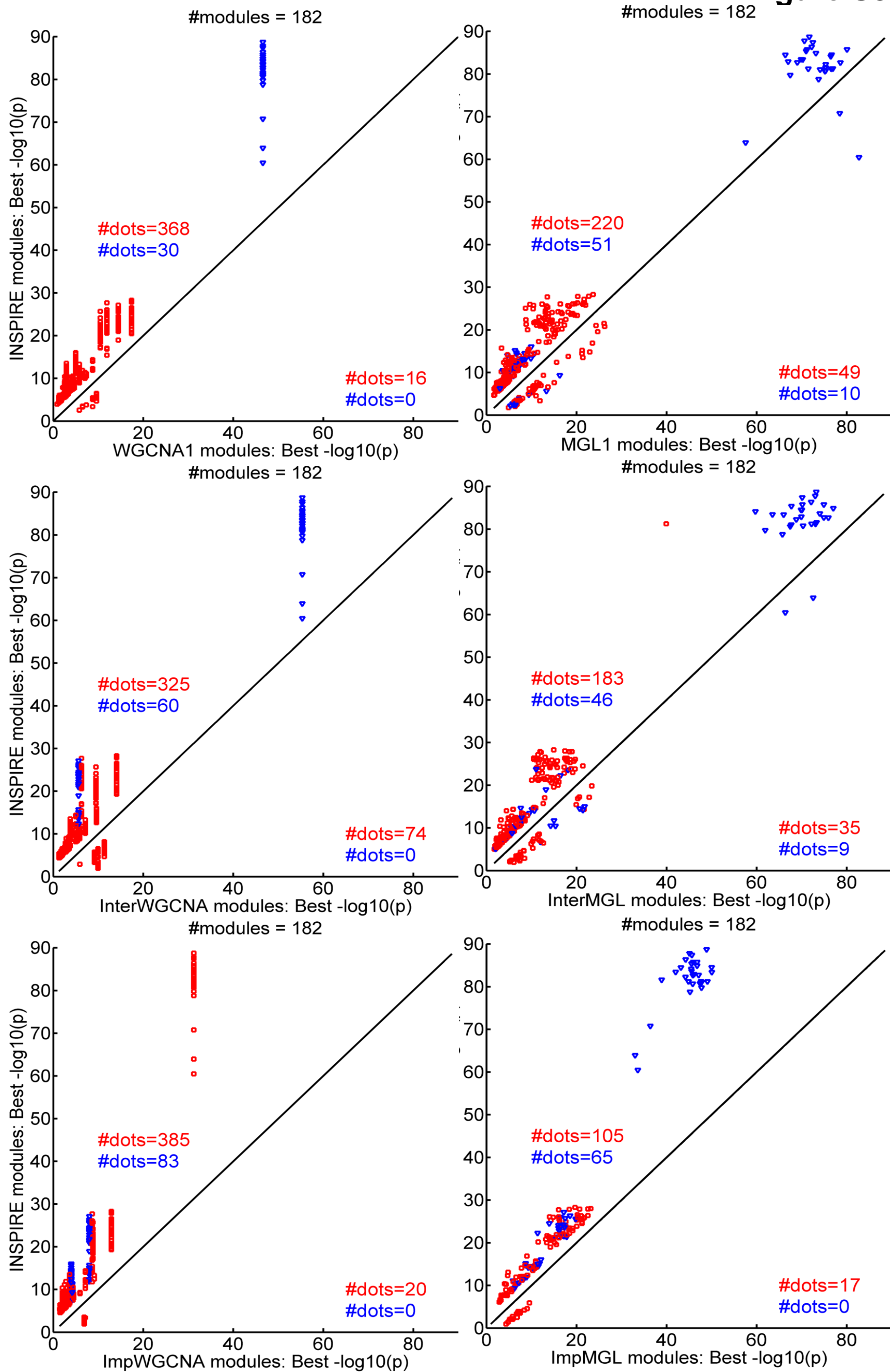

Supplement: Additional file 7: Figure S5. — A For k = 182, the best − log10 p from the functional enrichment of the modules learned by each of the six competing methods (on the x-axis) are compared to the best − log10 p from the functional enrichment of the modules learned by INSPIRE (on the y-axis). Each dot corresponds to a KEGG, Reactome, or BioCarta GeneSet and only the GeneSets with a Bonferroni corrected Fisher’s exact test p <0.05 in at least one of the compared two methods are shown on each plot. For MGL variants and INSPIRE, the results from multiple runs are shown on each plot. We only considered the GeneSets that show sufficiently different levels of significance, i.e. | log10 p(i) − log10 p(m)| ≥ δ, where “i” means INSPIRE and “m” means the alternative method. δ = 6 here and the results were consistent for varying δ. B For k = 182, the best − log10 p from the ChEA enrichment of the modules learned by each of the six competing methods (on the x-axis) are compared to the best − log10 p from the ChEA enrichment of the modules learned by INSPIRE (on the y-axis). Each dot is for a group of genes composed of a TF and its targets and only the TFs with a Bonferroni corrected Fisher’s exact test p <0.05 in at least one of the compared two methods are shown on each plot. For MGL variants and INSPIRE, the results from multiple runs are shown on each plot. We only considered the TFs that show sufficiently different levels of significance, i.e. | log10 p(i) − log10 p(m)| ≥ δ, where “i” means INSPIRE and “m” means the alternative method. We set δ = 3 here and the results were consistent for varying δ. Blue dots represent the TFs which are contained by the INSPIRE module which was significantly enriched for the target genes of that TF; red dots represent the TFs which are contained by an INSPIRE module different than the INSPIRE module that was significantly enriched for the target genes of that TF. (PDF 738 kb) [file 13073_2016_319_MOESM7_ESM.pdf]

Figure S6A

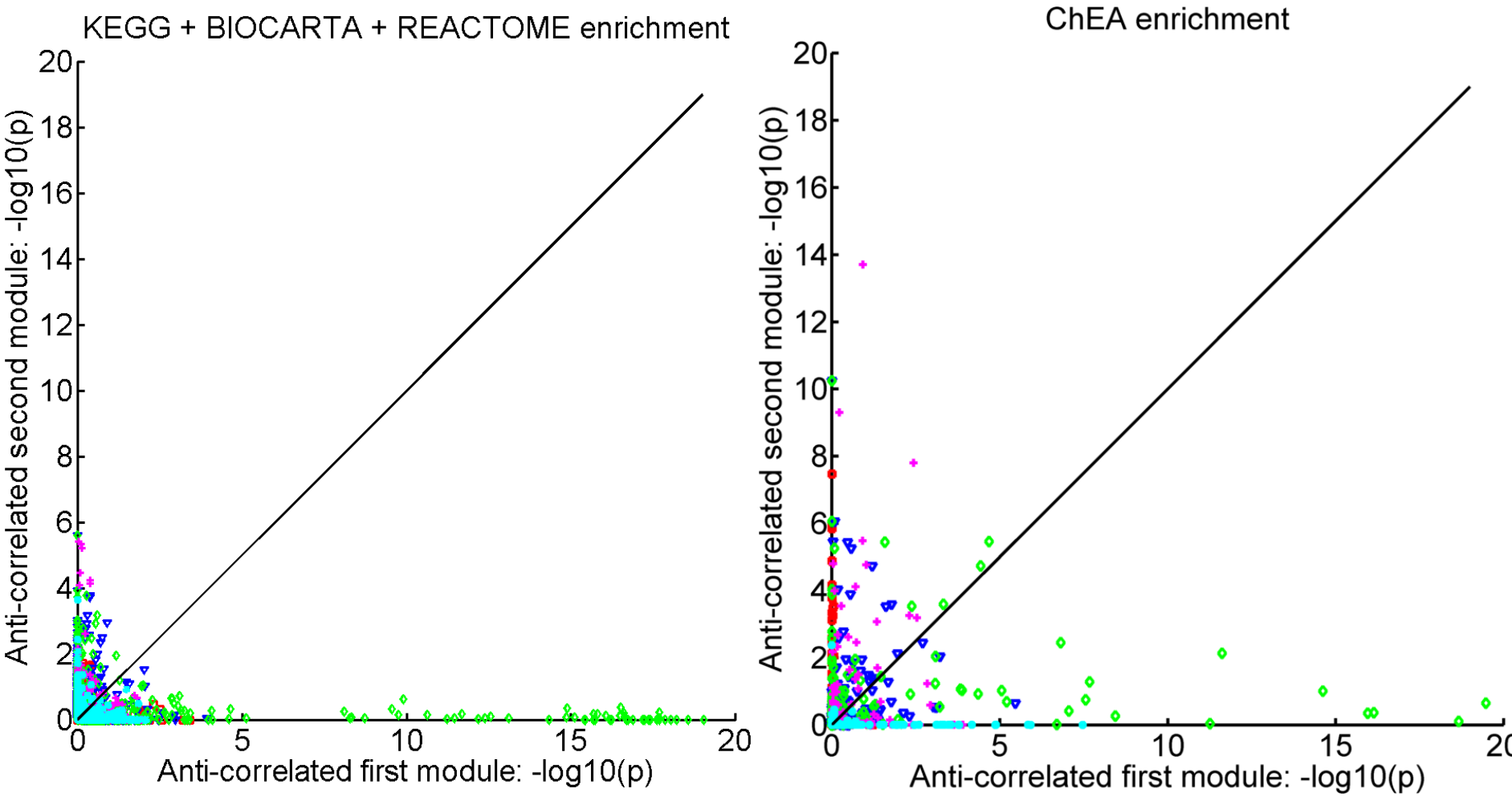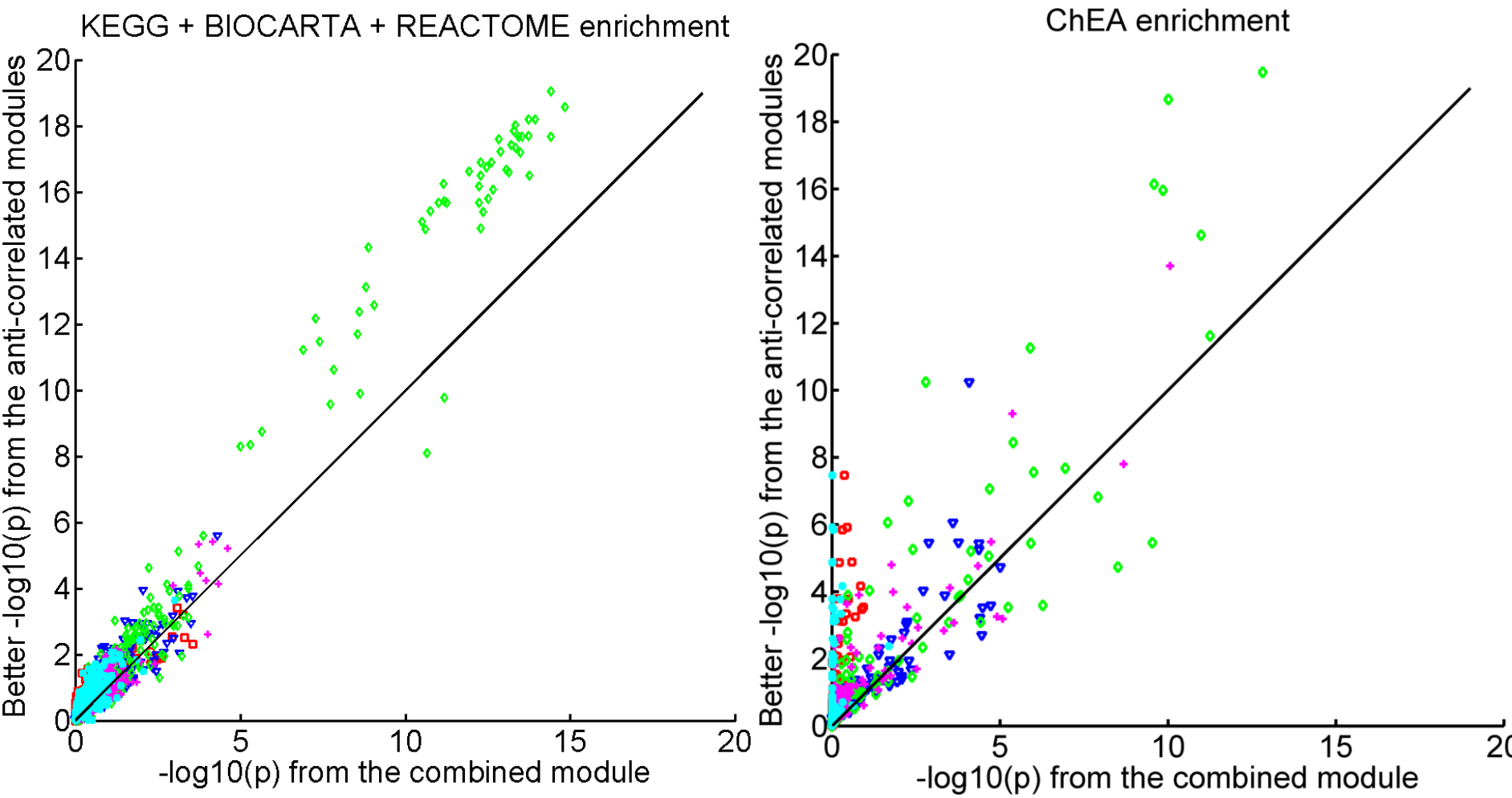

- modules 8 & 11: -0.85
- modules 12 & 47: -0.8
- modules 10 & 47: -0.8
- modules 7 & 50: -0.8
- modules 11 & 74: -0.75

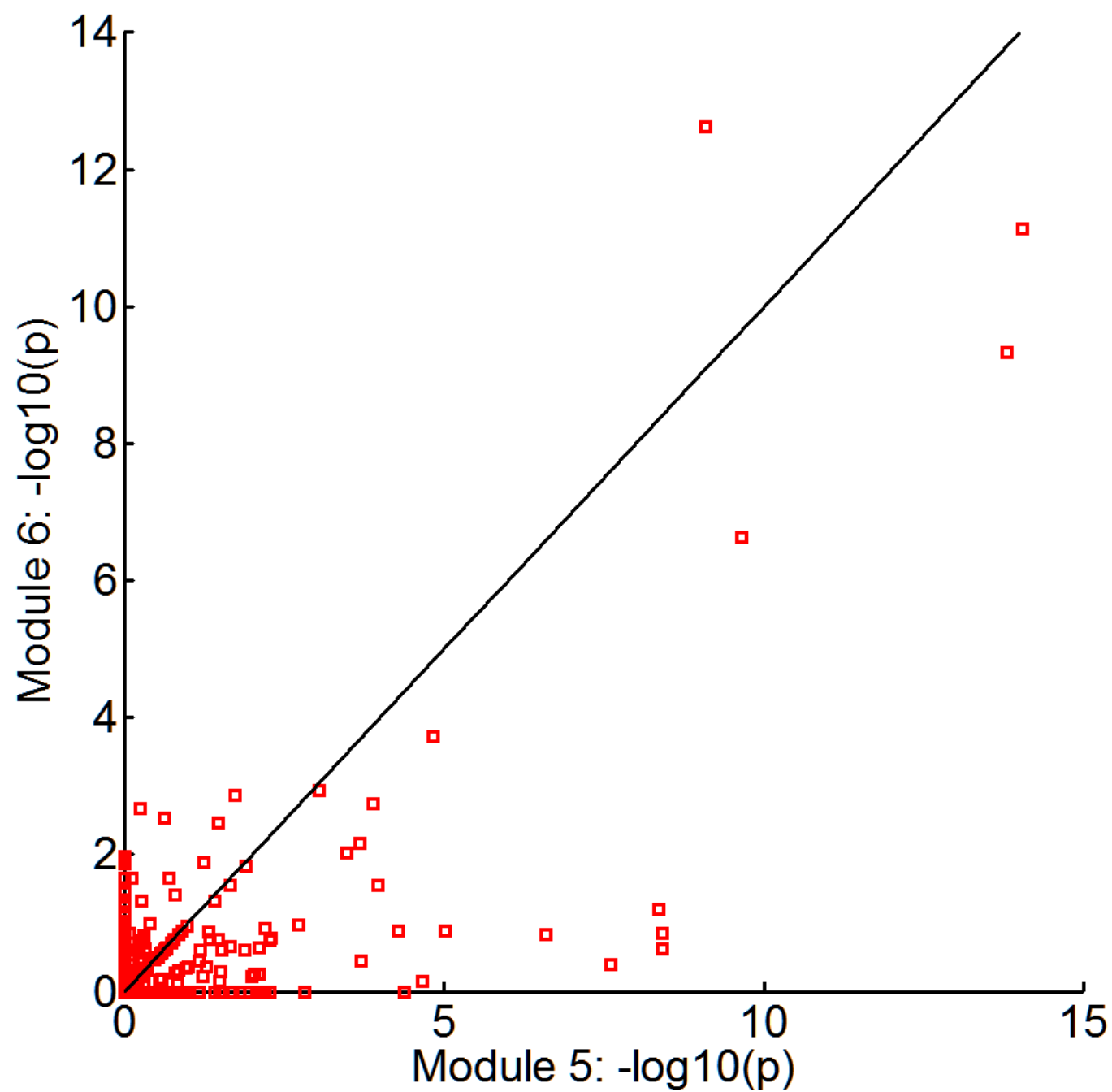

ChEA enrichment

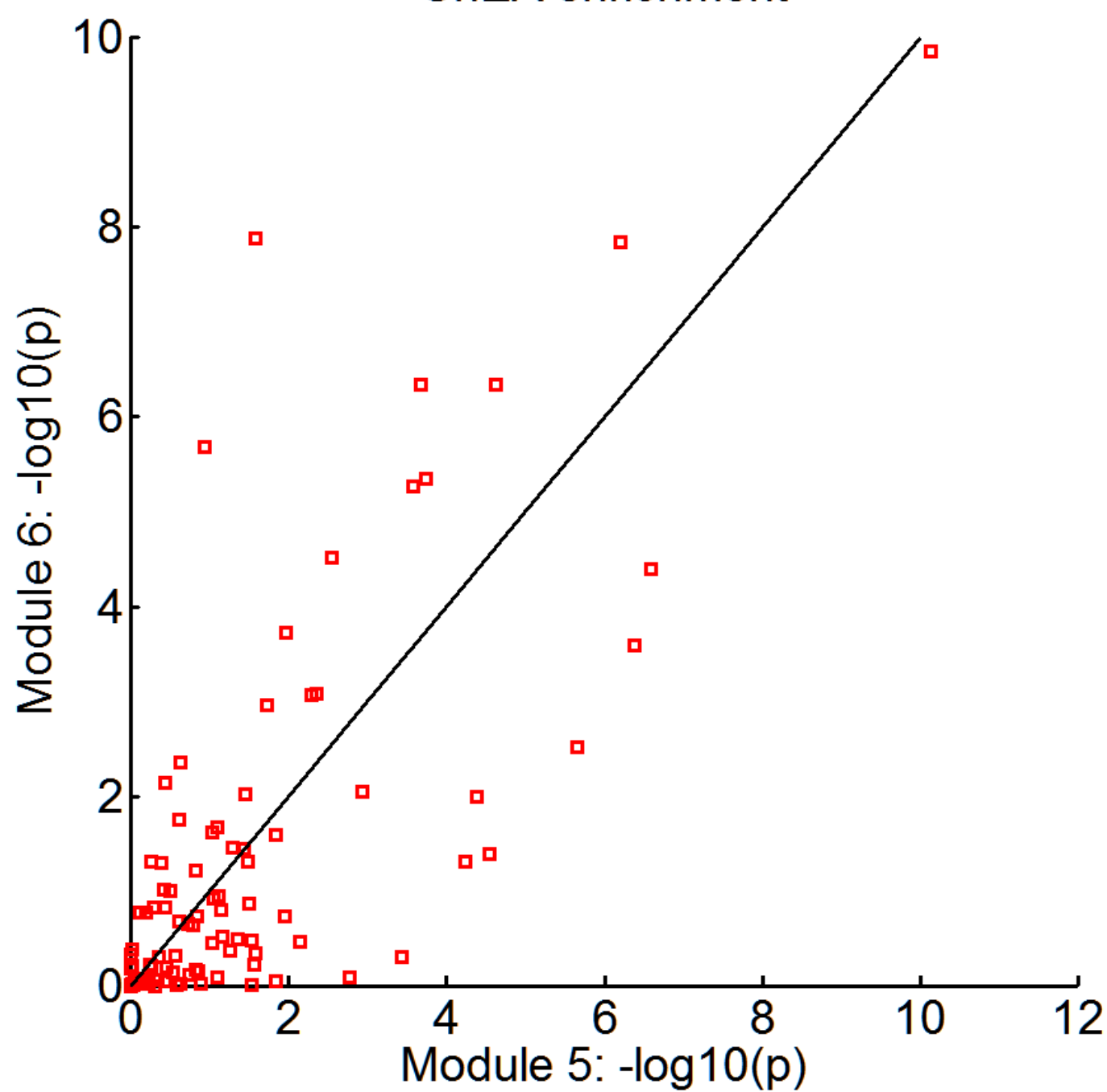

Supplement: Additional file 10: Figure S6. — A − log10 p from the KEGG, Reactome, or BioCarta GeneSet enrichment (left) and from the TF binding enrichment (right) is compared for five negatively correlated module pairs with the biggest absolute correlation in the nine-dataset experiment. Each one of the two negatively correlated modules is shown on one of the x-axis or y-axis, and each dot corresponds to a KEGG, Reactome, or BioCarta GeneSet (left) or a group of genes composed of a TF and its targets (right). B − log10 p from the KEGG, Reactome, or BioCarta GeneSet enrichment (top) and from the TF binding enrichment (bottom) is compared for module 5 (on the x-axis) and 6 (on the y-axis). Each dot corresponds to a KEGG, Reactome, or BioCarta GeneSet (left) or a group of genes composed of a TF and its targets (right). (PDF 194 kb) [file 13073_2016_319_MOESM10_ESM.pdf]

Figure S7A

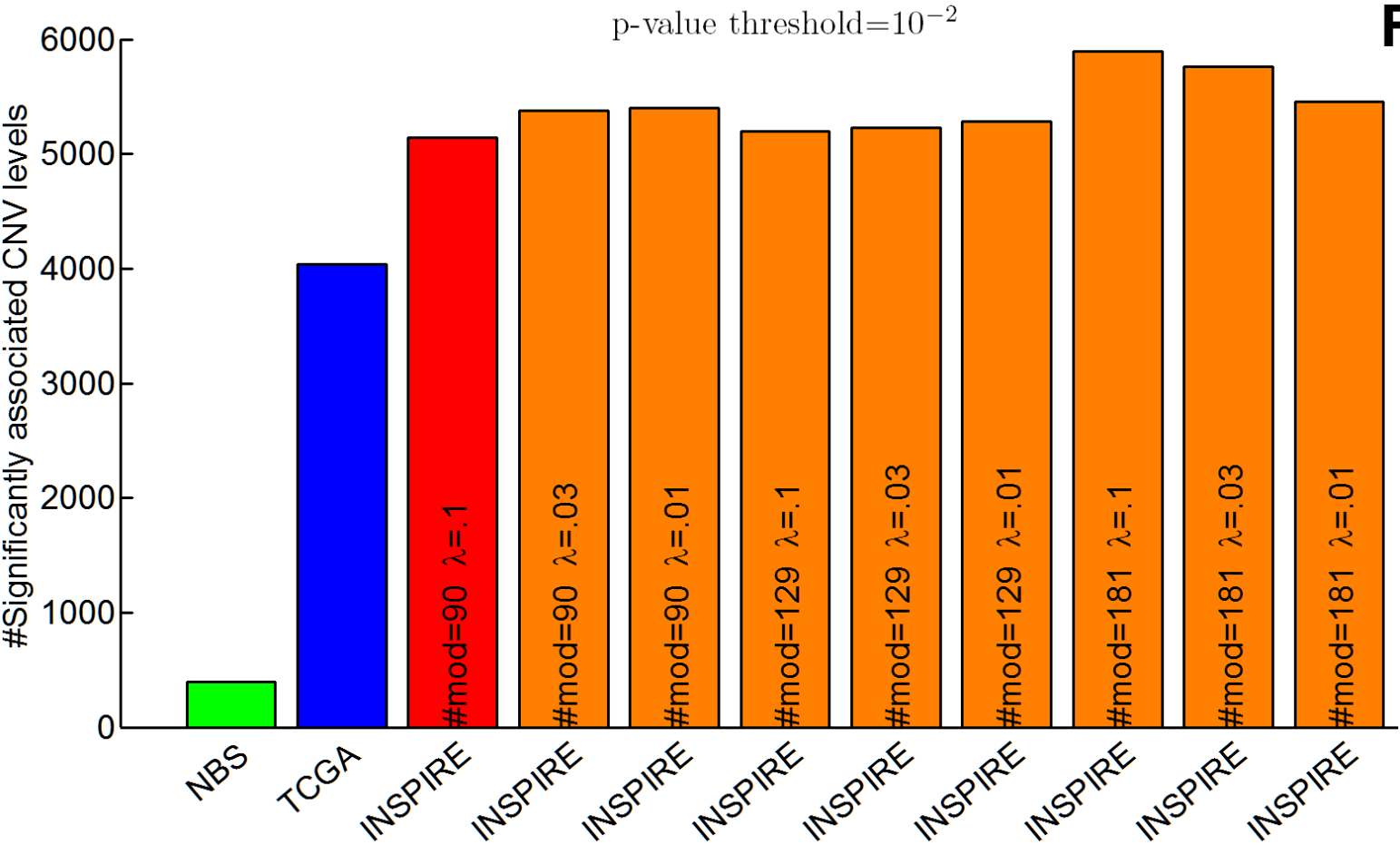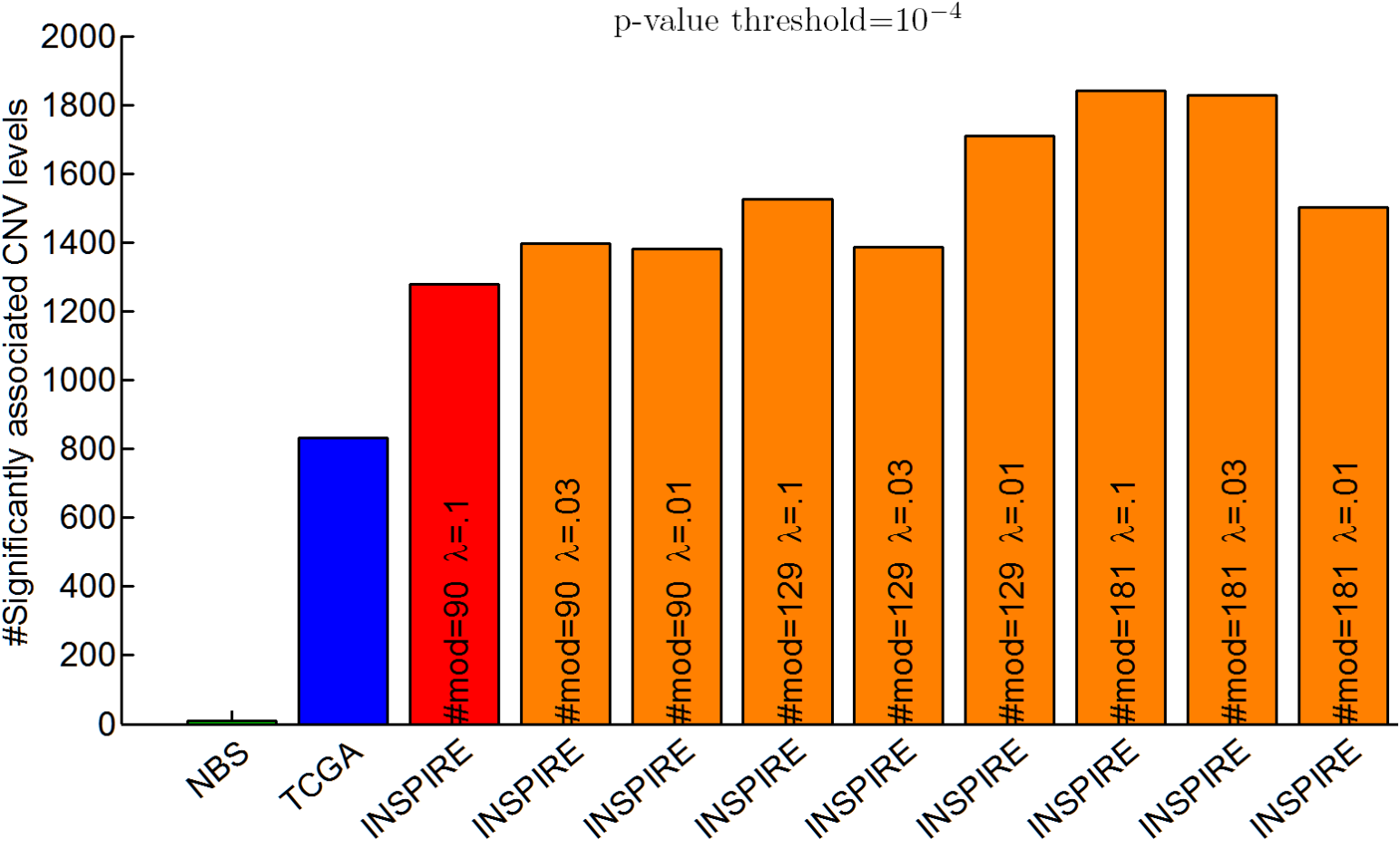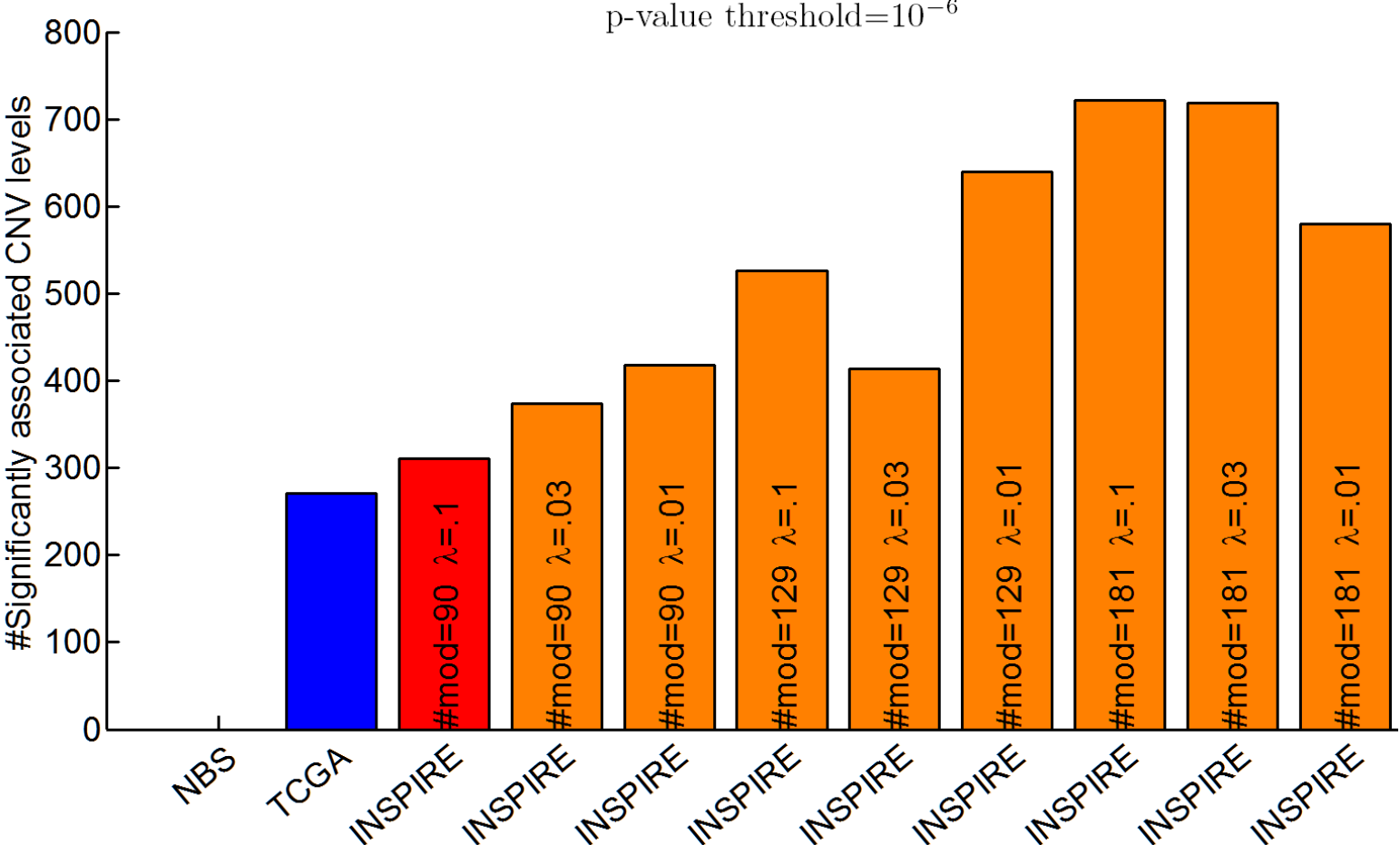

Figure S7B

(i)

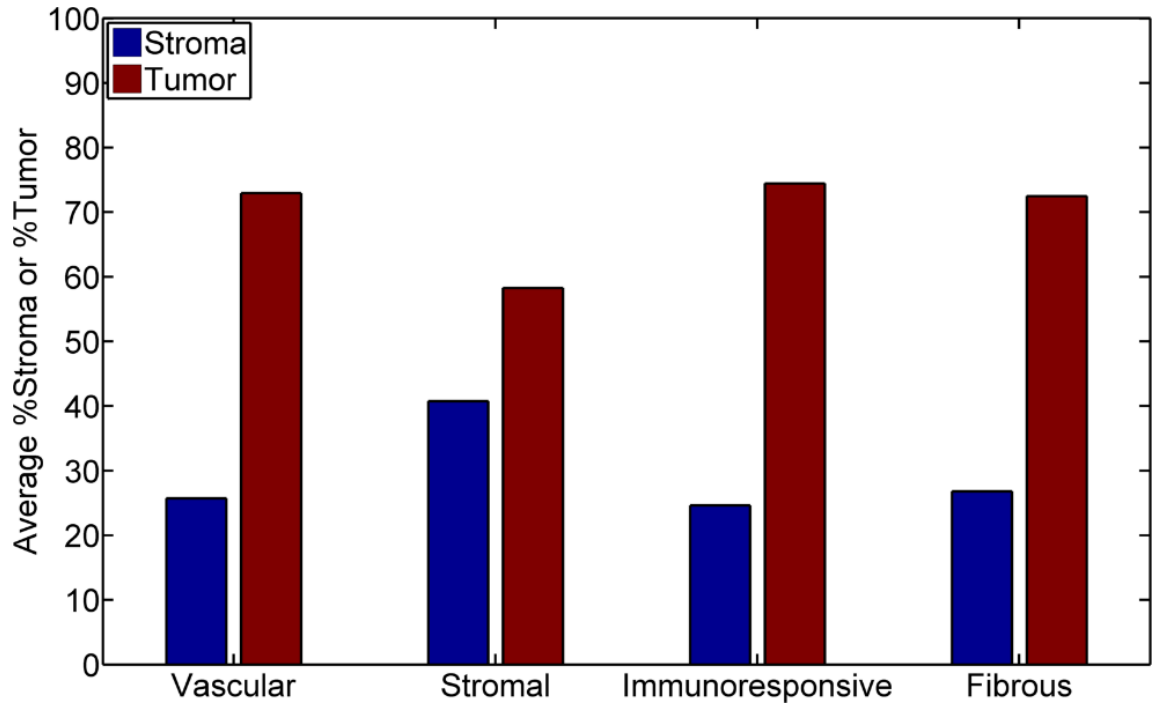

(ii)

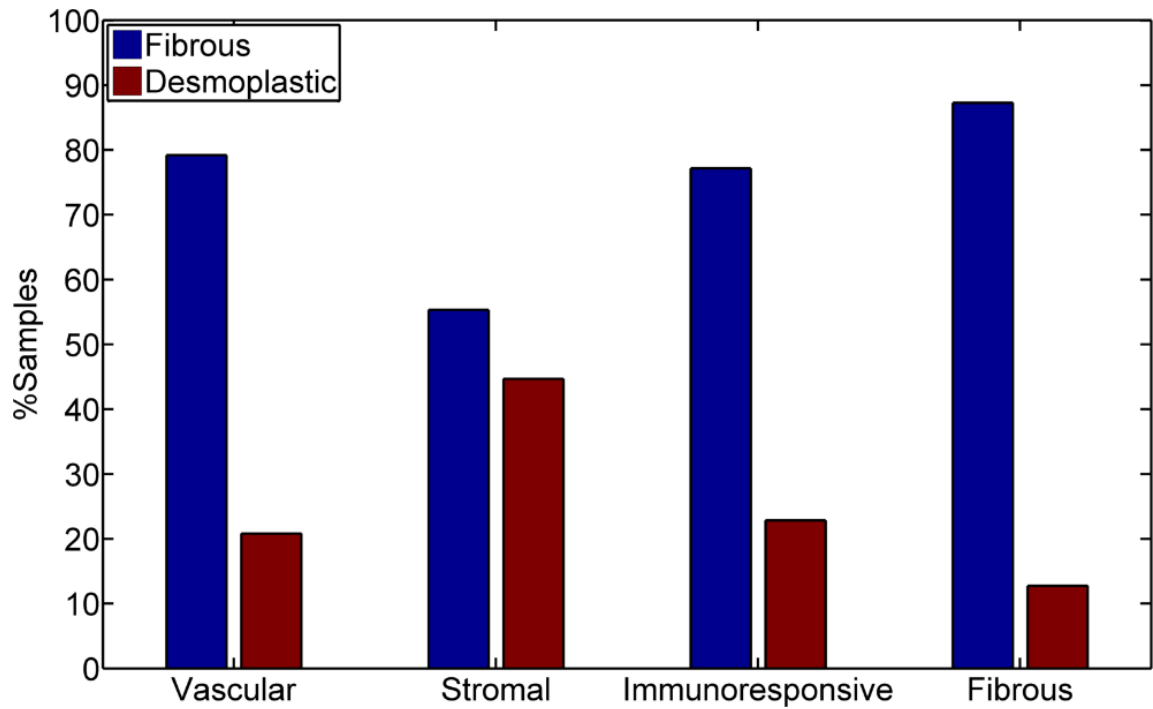

(iii)

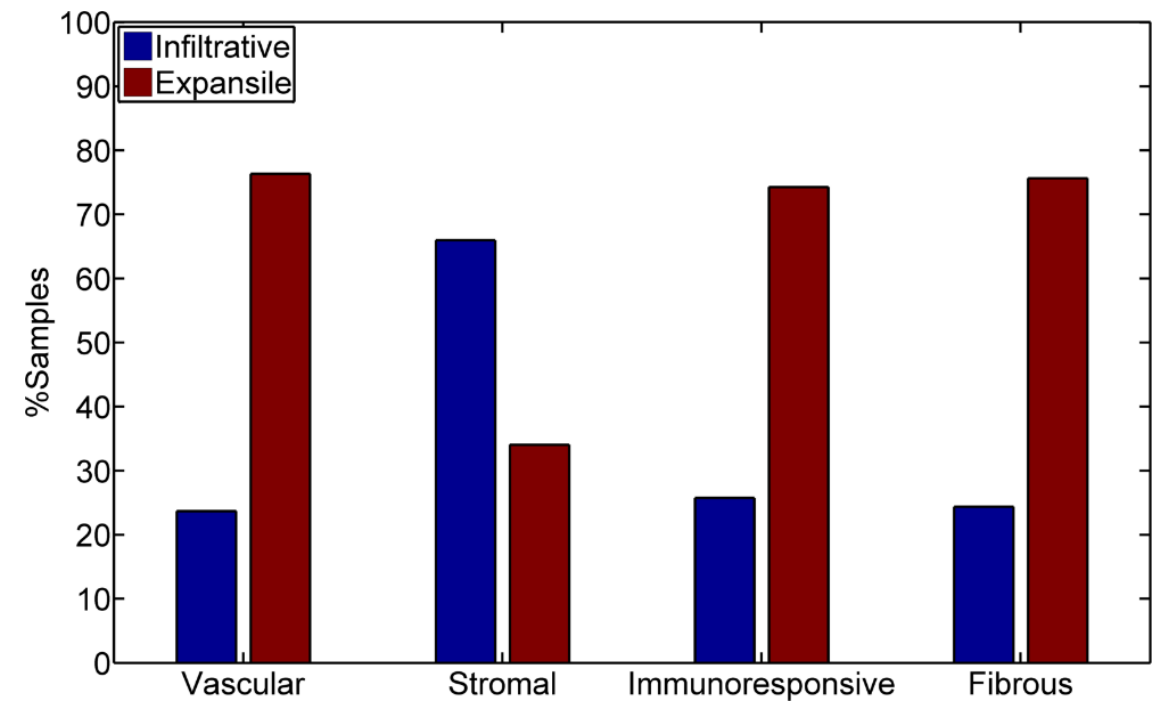

(iv)

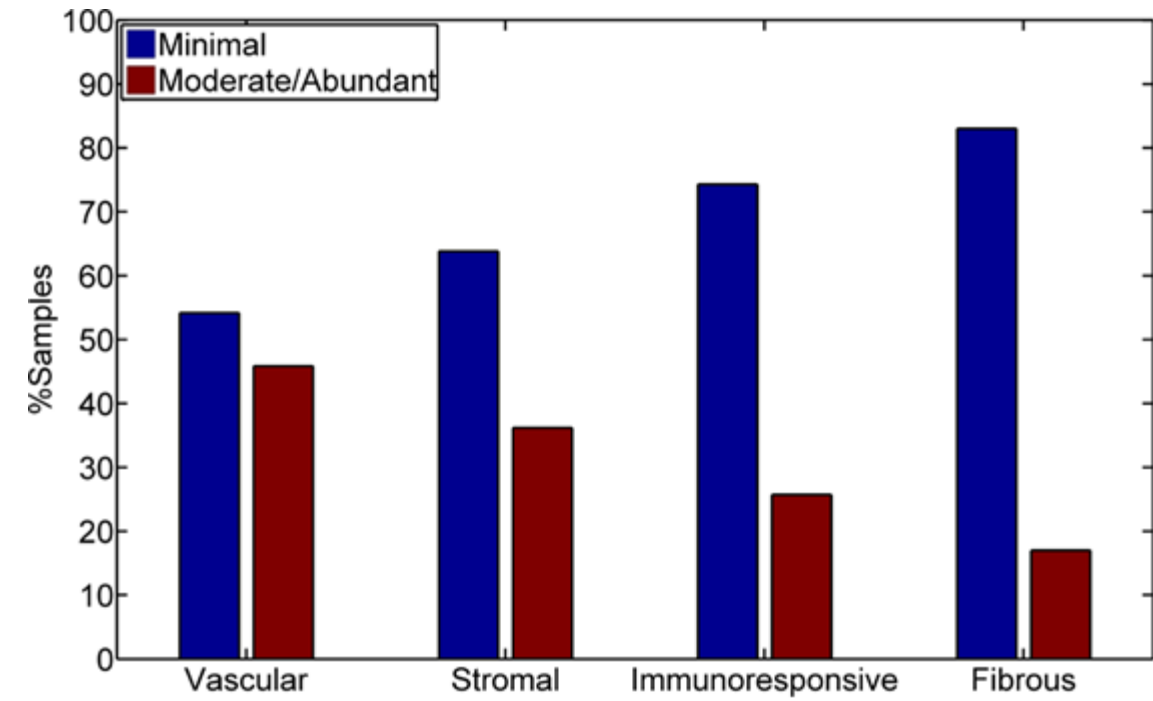

Supplement: Additional file 16: Figure S7. — A For three different Pearson’s correlation p value thresholds (10–2,10–4, 10–6, respectively, from top to bottom), the number of genes whose CNV levels are significantly associated with the learned ovarian cancer subtypes are shown for three methods: (1) the subtypes learned by a method that uses mutation profiles for the network-based stratification (NBS) [60] method (green); (2) the subtypes inferred from TCGA study [23] (blue); and (3) INSPIRE with varying sparsity tuning parameters (orange or red). Each bar for INSPIRE represents a setting with a different module count (k) and module network sparsity parameter (λ). The red bar for INSPIRE corresponds to the setting on which our biological analysis is based. B (1) For each of the INSPIRE subtypes, the percent stroma (blue bar) and the percent tumor (red bar) averaged over the patients in the subtype are shown; (2) for each of the INSPIRE subtypes, the percentage of the patients in the subtype with fibrous stroma (blue bar) and desmoplastic stroma (red bar) are shown; (3) for each of the INSPIRE subtypes, the percentage of the patients in the subtype with infiltrative invasion pattern (blue bar) and expansile invasion pattern (red bar) are shown; (4) for each of the INSPIRE subtypes, the percentage of the patients in the subtype with minimal vessels (blue bar) and moderate or abundant vessels (red bar) are shown. (PDF 379 kb) [file 13073_2016_319_MOESM16_ESM.pdf]

Figure S8

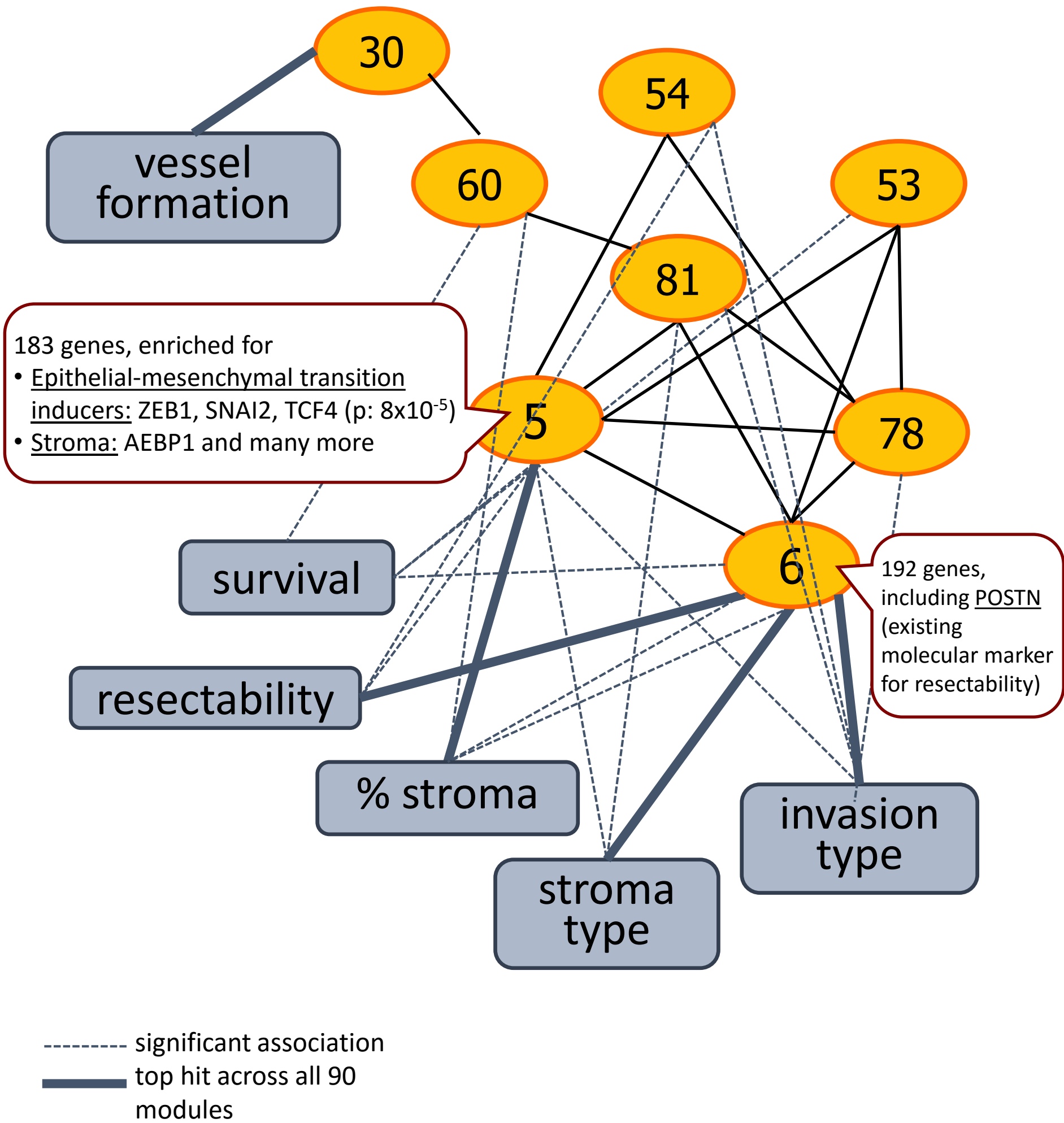

Supplement: Additional file 20: Figure S8. — The interactions among the modules that show significant correlations with the important phenotypes in the TCGA ovarian cancer data, as shown by red bars in Fig. 5a. The edges are shown by black lines. Also, as a recap of Fig. 5a for those specific modules, the significant associations of each module with six important phenotypes in ovarian cancer are shown by dotted blue lines and the associations that are the most significant among all modules are shown by solid blue lines. The details for modules 5 and 6, which achieve top hits for a total of four phenotypes, are given as well. (PDF 248 kb) [file 13073_2016_319_MOESM20_ESM.pdf]

Figure S9A

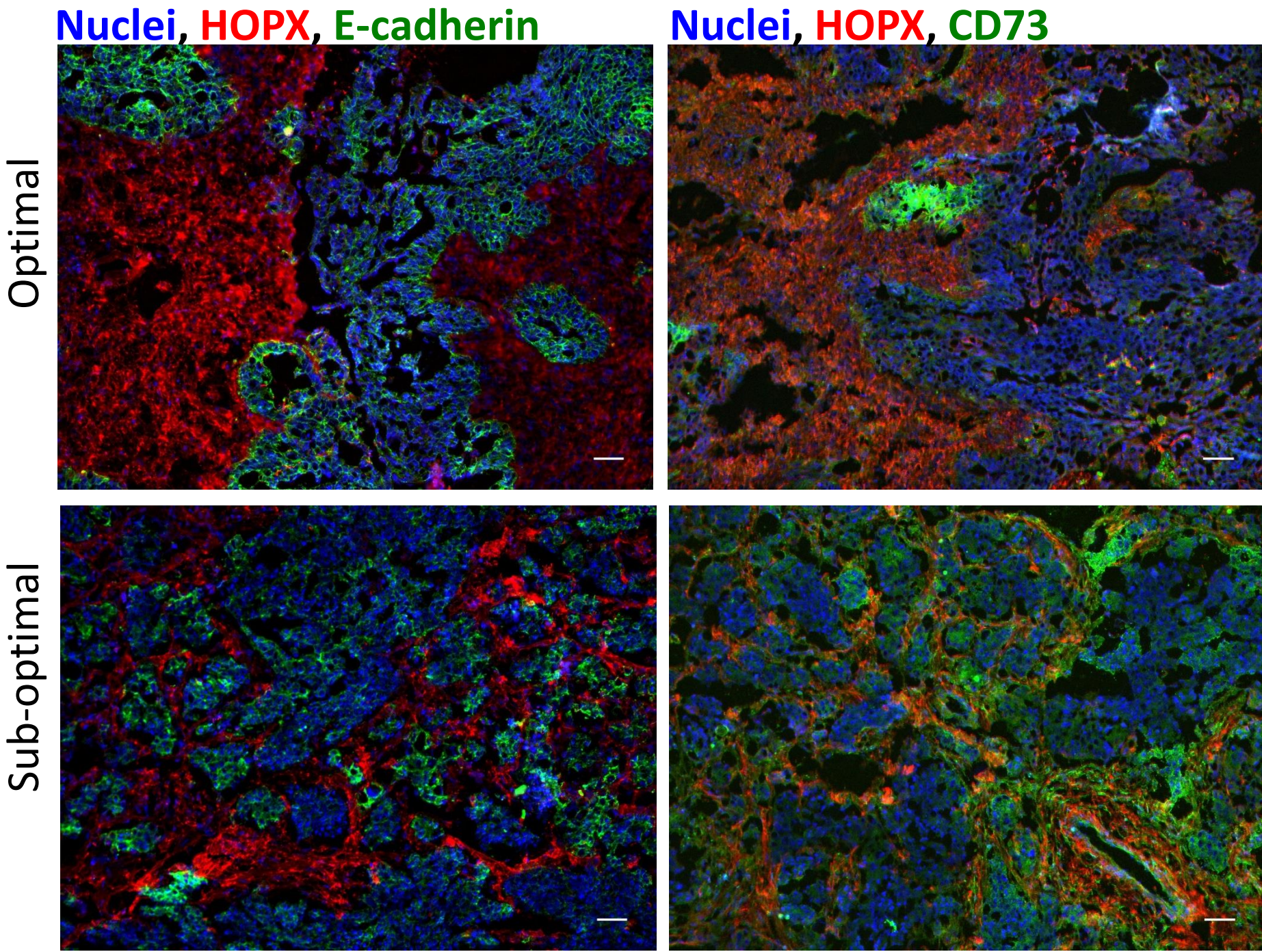

Figure S9B

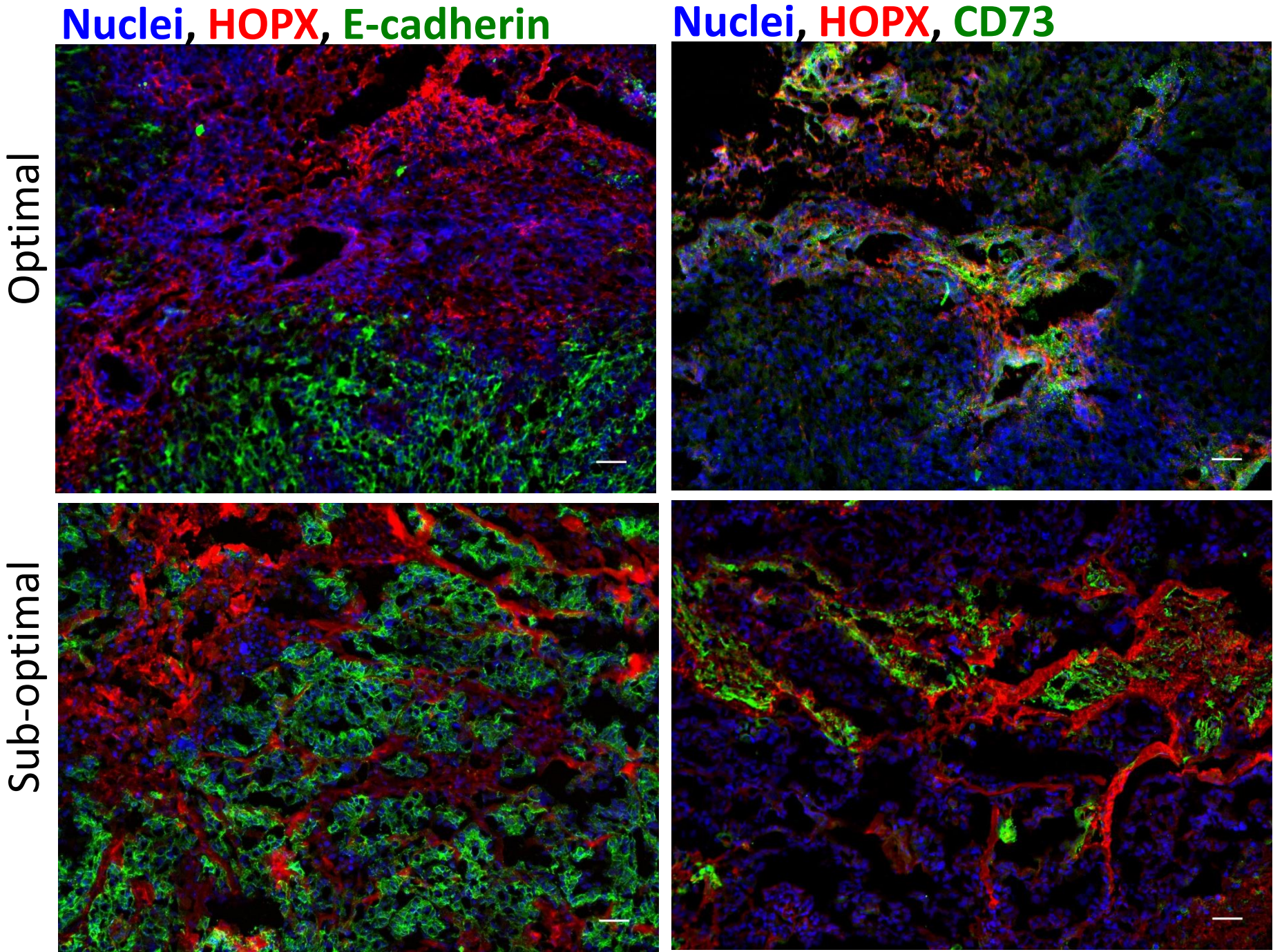

(i)

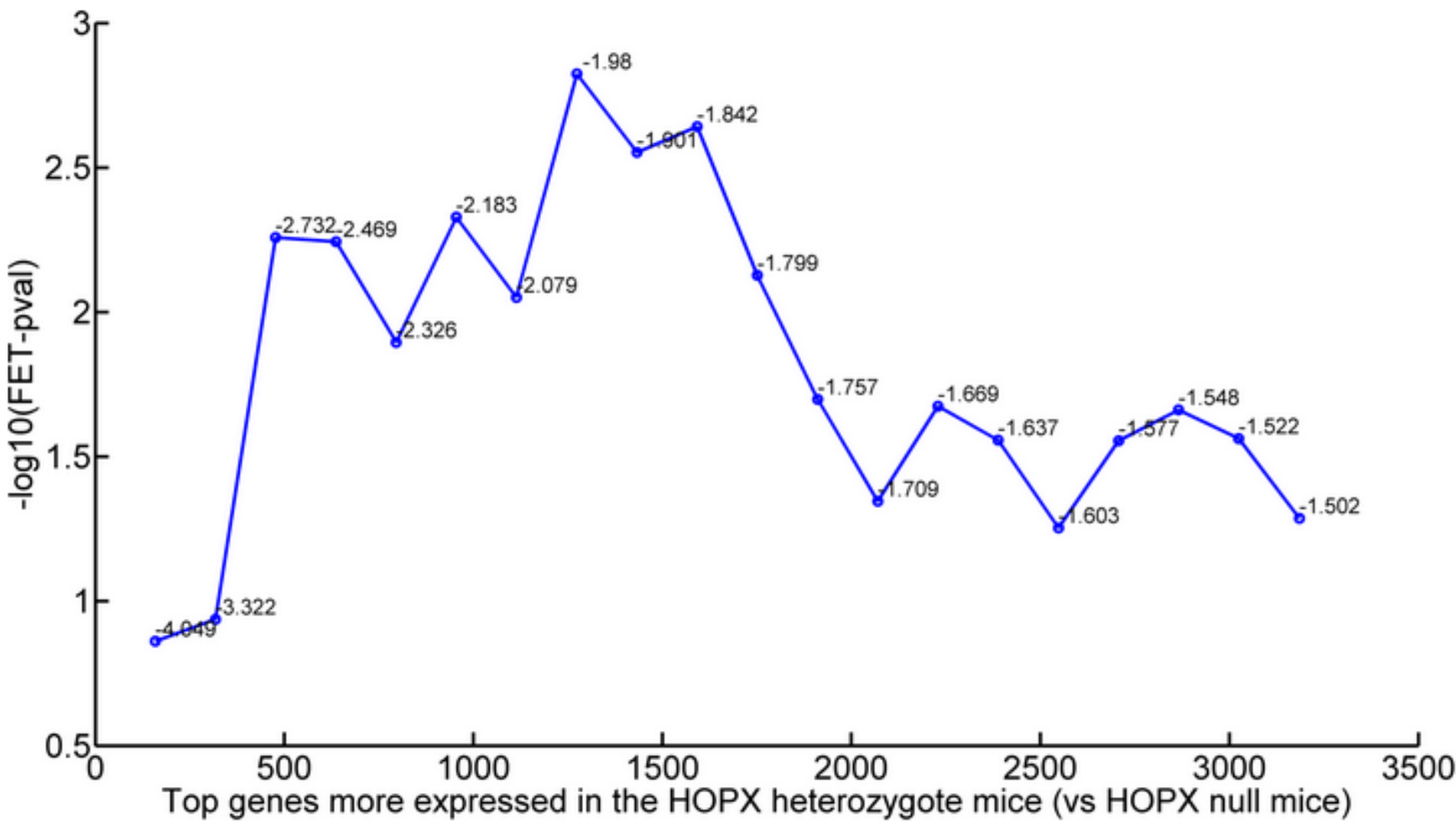

(ii)

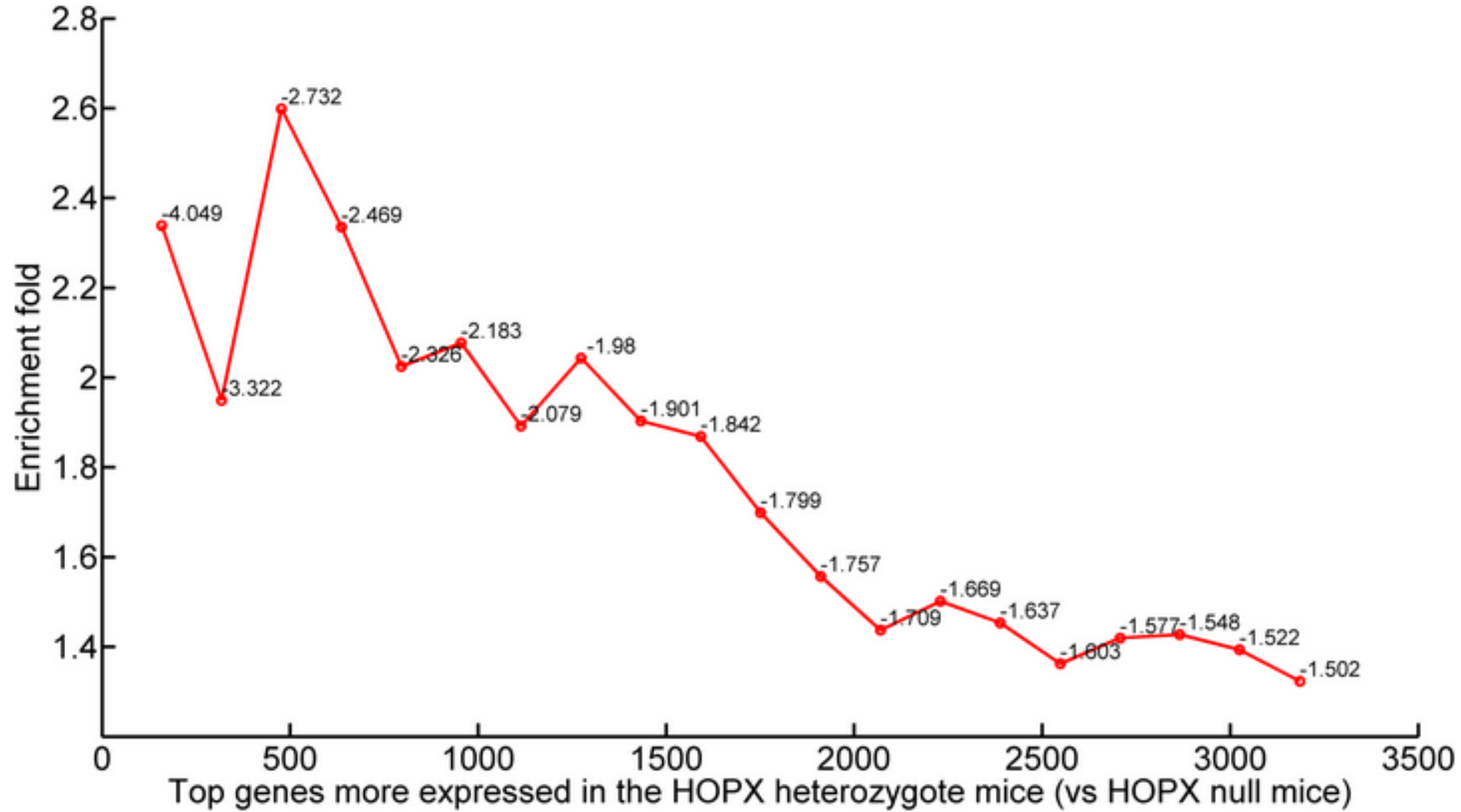

(i)

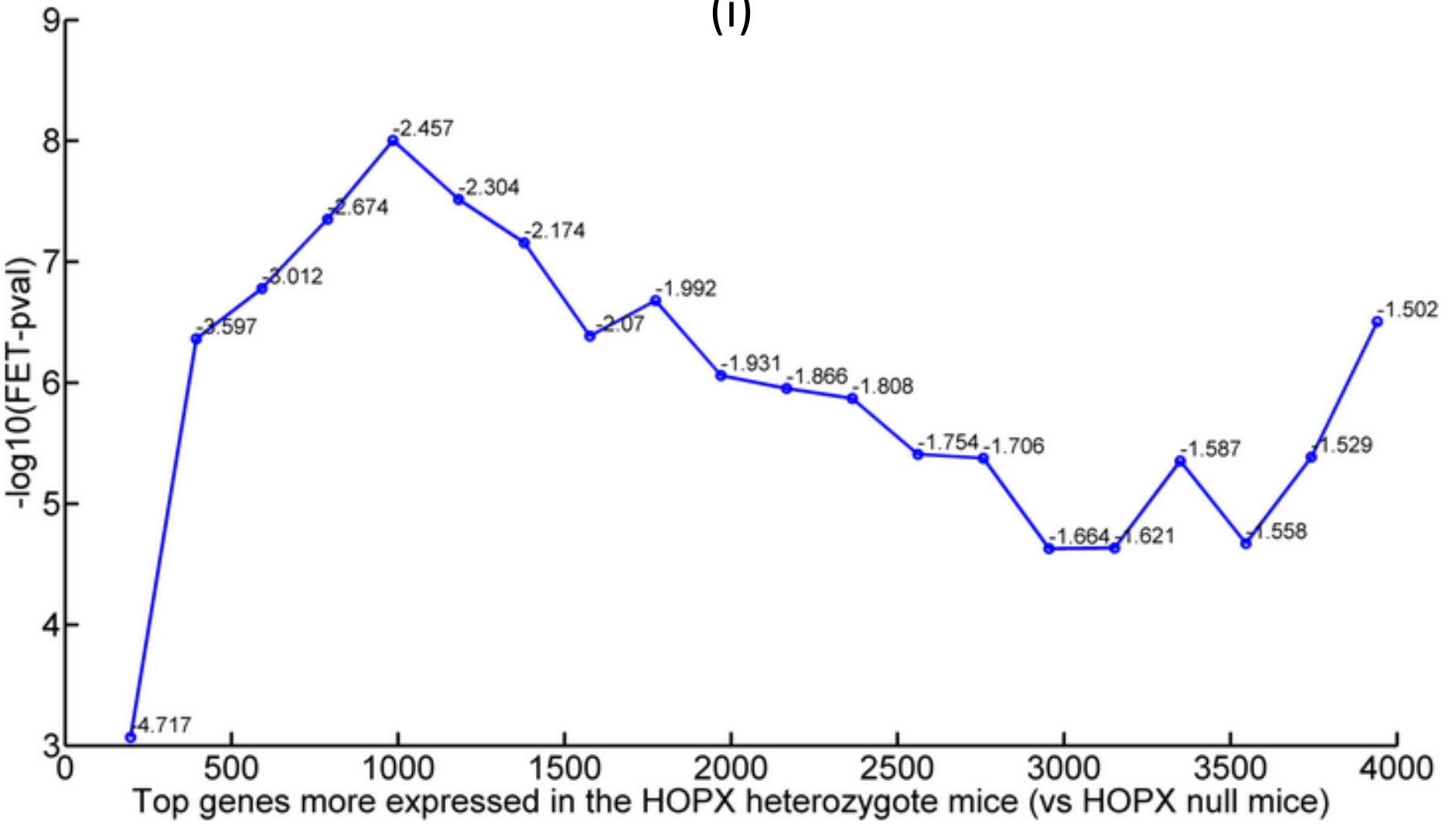

(ii)

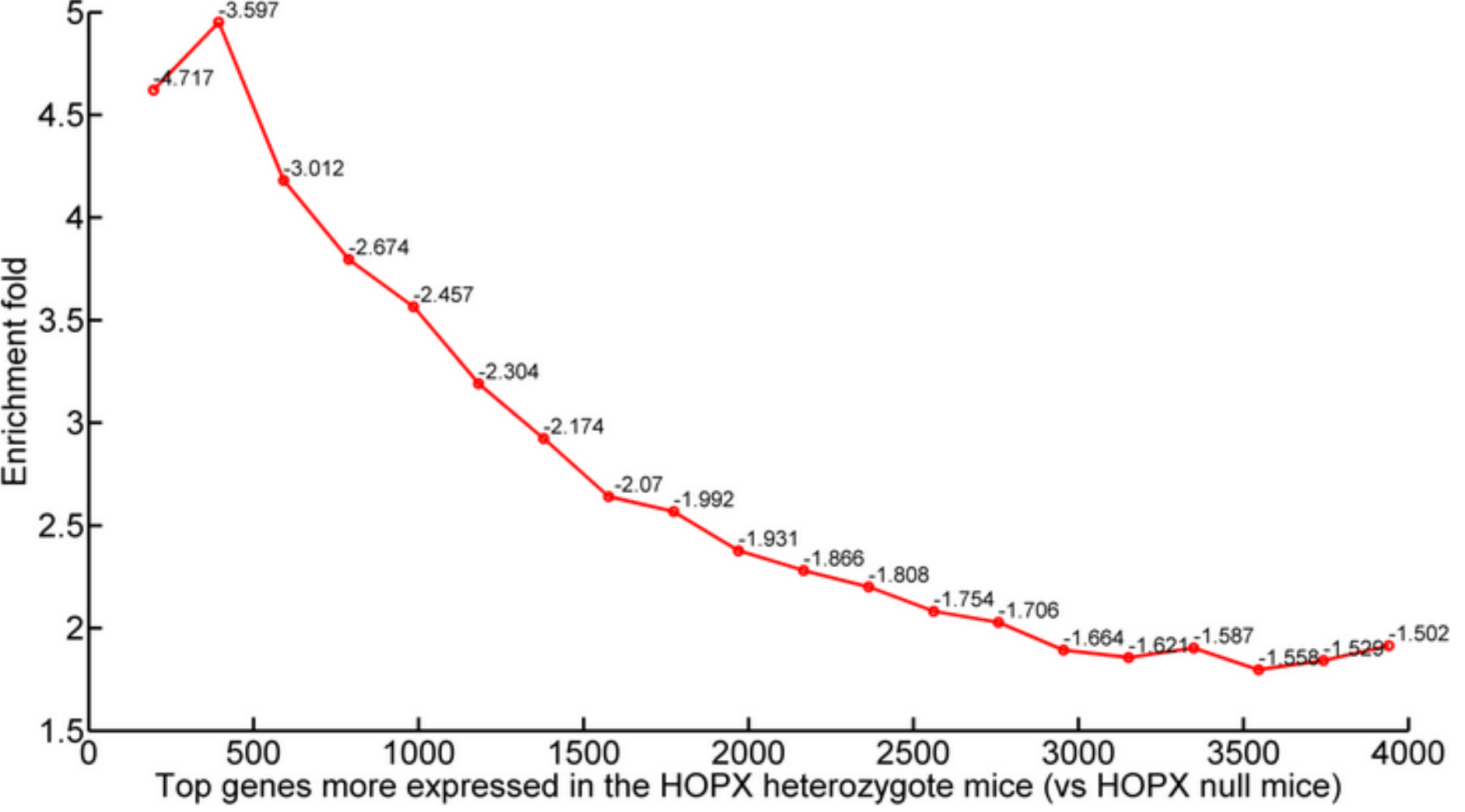

Supplement: Additional file 22: Figure S9. — A, B Additional fluorescent images of ovarian tumors from sub-optimally debulked and optimally debulked patients. Each row is a patient. As in Fig. 6b, HOPX is localized to the stroma and does not overlap with E Cadherin positive cancer cells. HOPX does however overlap with CD73, a MSC marker. C The functional enrichment p value (i) and the fold enrichment (ii) of module 5 genes for the genes downregulated in Hopx-null mice for different thresholds of fold-change in expression. The corresponding fold-change threshold is displayed next to each dot on the curves. The x-axes represent the number of downregulated genes in Hopx-null mice whose expression fold-change passes the fold-change threshold displayed next to the corresponding value on the curve. D The functional enrichment p value (i) and the fold enrichment (ii) of module 5 genes for the genes downregulated in Hopx-null mice upon inhibition of Wnt signaling for different thresholds of fold-change in expression. The corresponding fold-change threshold is displayed next to each dot on the curves. The x-axes represent the number of downregulated genes in Hopx-null mice upon Wnt inhibition whose expression fold-change passes the fold-change threshold displayed next to the corresponding value on the curve. (PDF 1484 kb) [file 13073_2016_319_MOESM22_ESM.pdf]
