# Supplementary material for: Extracting a low-dimensional description of multiple gene expression datasets reveals a potential driver for tumor-associated stroma in ovarian cancer
Source: Genome Med. 2016 Jun 10;8:66. doi: 10.1186/s13073-016-0319-7 (PMC4902951; doi:10.1186/s13073-016-0319-7)
Supplement: Additional file 6: Table S2. — The two ovarian cancer gene expression datasets we used in the second set of experiments. (DOC 27 kb) [file 13073_2016_319_MOESM6_ESM.doc]

**Table S2 The two ovarian cancer gene expression datasets we used in the second set of experiments.**

| **Name** | **Samples (n)** | **Genes (n)** | **Platform** |
| --- | --- | --- | --- |
| GSE19829.GPL570 | 28 | 18,113 | Affymetrix HGU133Plus2 |
| GSE19829. [GPL8300](http://www.ncbi.nlm.nih.gov/geo/query/acc.cgi?acc=GPL8300) | 42 | 8331 | Affymetrix HGU95Av2 |
