# Supplementary material for: Extracting a low-dimensional description of multiple gene expression datasets reveals a potential driver for tumor-associated stroma in ovarian cancer
Source: Genome Med. 2016 Jun 10;8:66. doi: 10.1186/s13073-016-0319-7 (PMC4902951; doi:10.1186/s13073-016-0319-7)
Supplement: Additional file 12: Table S6. — The − log10 p from the pathway (KEGG, Reactome, and BioCarta) enrichment test (top) and from the TF binding enrichment test (bottom) are compared between modules 5 and 6. We show the five pathways or TFs that have the largest difference in the value of − log10 p between the two modules. (DOC 48 kb) [file 13073_2016_319_MOESM12_ESM.doc]

**Table S6** The
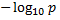
from the pathway (KEGG, Reactome, and BioCarta) enrichment test (top) and from the TF binding enrichment test (bottom) are compared between modules 5 and 6. We show the five pathways or TFs that have the largest difference in the value of
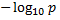
 between the two modules.

|  |  | **Module 5** | **Module 6** |
| --- | --- | --- | --- |
| **GeneSet** | REACTOME_SMOOTH_MUSCLE_CONTRACTION | 8.4036 | 0.6308 |
|  | REACTOME_SIGNALING_BY_PDGF | 8.41 | 0.8542 |
|  | REACTOME_MUSCLE_CONTRACTION | 7.604 | 0.3971 |
|  | REACTOME_NCAM1_INTERACTIONS | 8.358 | 1.1928 |
|  | REACTOME_NCAM_SIGNALING_FOR_NEURITE_OUT_GROWTH | 6.5945 | 0.8311 |
| **TF** | PAX3-FKHR | 1.5796 | 7.8889 |
|  | POU3F2 | 0.9312 | 5.6912 |
|  | TP53 | 4.5343 | 1.3829 |
|  | AR | 5.6507 | 2.5125 |
|  | TRIM28 | 3.4385 | 0.3065 |
