# Supplementary material for: Extracting a low-dimensional description of multiple gene expression datasets reveals a potential driver for tumor-associated stroma in ovarian cancer
Source: Genome Med. 2016 Jun 10;8:66. doi: 10.1186/s13073-016-0319-7 (PMC4902951; doi:10.1186/s13073-016-0319-7)
Supplement: Additional file 13: Table S7. — For module 5, module 6, and a hypothetical module containing all genes in modules 5 and 6, the prediction accuracy is compared in six prediction tasks via CV tests. The best performance for each prediction task is highlighted in green. (DOC 31 kb) [file 13073_2016_319_MOESM13_ESM.doc]

**Table S7** For module 5, module 6, and a hypothetical module containing all genes in modules 5 and 6, the prediction accuracy is compared in six prediction tasks via CV tests. The best performance for each prediction task is highlighted in orange.

|  | **Module 5** | **Module 6** | **Modules 5 and 6 merged** |
| --- | --- | --- | --- |
| Percent stroma | 0.8242 | 0.8063 | 0.818 |
| Stroma type | 0.8551 | 0.8634 | 0.86 |
| Vessels | 0.7903 | 0.7913 | 0.7892 |
| Invasion | 0.8382 | 0.847 | 0.8458 |
| Residual tumor | 0.8376 | 0.8397 | 0.8386 |
| Survival | 0.5465 | 0.5406 | 0.5457 |
