# Supplementary material for: Extracting a low-dimensional description of multiple gene expression datasets reveals a potential driver for tumor-associated stroma in ovarian cancer
Source: Genome Med. 2016 Jun 10;8:66. doi: 10.1186/s13073-016-0319-7 (PMC4902951; doi:10.1186/s13073-016-0319-7)
Supplement: Additional file 14: Table S8. — We remove 20 %, 40 %, 60 %, and 80 % of the genes in each module whose expression levels are least significantly associated with the respective phenotype and regenerate latent variables from the rest of the genes in the modules. For each of those settings, the prediction performance is compared in six prediction tasks via CV tests. The best performance(s) for each prediction task is highlighted in green. (DOC 33 kb) [file 13073_2016_319_MOESM14_ESM.doc]

**Table S8** We remove 20%, 40%, 60%, and 80% of the genes in each module whose expression levels are least significantly associated with the respective phenotype and regenerate latent variables from the rest of the genes in the modules. For each of those settings, the prediction performance is compared in six prediction tasks via CV tests. The best performance(s) for each prediction task is highlighted in green.

|  | **Original** | **20% removed** | **40% removed** | **60% removed** | **80% removed** |
| --- | --- | --- | --- | --- | --- |
| Percent stroma | 0.7951 | 0.7908 | 0.7712 | 0.7725 | 0.7591 |
| Stroma type | 0.8367 | 0.7857 | 0.7959 | 0.7959 | 0.8367 |
| Vessels | 0.7634 | 0.7419 | 0.7204 | 0.6989 | 0.6882 |
| Invasion | 0.7717 | 0.7609 | 0.75 | 0.7391 | 0.7174 |
| Residual tumor | 0.7912 | 0.7912 | 0.7842 | 0.7773 | 0.768 |
| Survival | 0.5689 | 0.5305 | 0.5302 | 0.5373 | 0.5459 |
