# Supplementary material for: Extracting a low-dimensional description of multiple gene expression datasets reveals a potential driver for tumor-associated stroma in ovarian cancer
Source: Genome Med. 2016 Jun 10;8:66. doi: 10.1186/s13073-016-0319-7 (PMC4902951; doi:10.1186/s13073-016-0319-7)
Supplement: Additional file 17: — INSPIRE-SupplementaryInformation. Supplementary notes. (DOC 94 kb) [file 13073_2016_319_MOESM17_ESM.doc]

**SUPPLEMENTARY NOTES**

**Supplementary Note 1: Genes whose CNV is associated with the subtypes**

**Stromal subtype:**

***Genes (association p value threshold is* 8 × 10^–5^*, gene count is 70):***

ABCB4, ACN9, AKAP9, ANKIB1, ASNS, BHLHA15, BRD4, C7ORF62, C8ORF37, CALB1, CALCR, CCNE2, CNBD1, CNGB3, CPNE3, CPQ, CSMD3, CYP51A1, DCAF4L2, DECR1, DLX5, DLX6.AS1, EIF3H, EPHX3, FKBP6, GATAD1, GEM, HEPACAM2, KRIT1, LAPTM4B, LMTK2, MTDH, MTERF, MTERFD1, NBN, NDUFAF6, NECAB1, NPTX2, OSGIN2, OTUD6B, PCLO, PEX1, PLEKHF2, PTDSS1, RAD21, RBM48, RIPK2, RUNX1T1, SDC2, SEMA3A, SEMA3D, SHFM1, SLC25A40, SLC26A7, SRI, STEAP1, STEAP2, TAC1, TECPR1, TMEM55A, TMEM64, TP53INP1, TRHR, TRIM50, TRIQK, TRPS1, TSPYL5, UQCRB, UTP23, YWHAG

***Functional enrichment of those genes (MSigDB C2 category, Bonferroni-corrected***
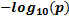
***):***

NIKOLSKY_BREAST_CANCER_8Q12_Q22_AMPLICON, 29.7339391253105

NIKOLSKY_BREAST_CANCER_7Q21_Q22_AMPLICON, 23.7934623738506

ONKEN_UVEAL_MELANOMA_UP, 2.59997036554253

**Vascular subtype:**

***Genes (association p value threshold is* 10^–12^*, gene count is 99):***

ADCK5, ADCY8, AGO2, ANXA13, ASAP1, ASAP1.IT1, BREA2, C8ORF31, CHRAC1, COL22A1, COLEC10, CPSF1, CYC1, CYHR1, CYP11B2, DENND3, DGAT1, EEF1D, ENPP2, EPPK1, EXOSC4, FAM135B, FAM49B, FAM83H, FAM84B, FAM91A1, FBXL6, FOXH1, GLI4, GPAA1, GPIHBP1, GPR20, GPT, GRINA, GSDMC, GSDMD, HHLA1, HPYR1, HSF1, KCNK9, KCNQ3, KHDRBS3, KIAA0196, KIAA1875, KIFC2, LINC00964, LOC731779, LRRC6, LY6E, LY6H, MAF1, MAPK15, MFSD3, MROH1, MROH6, MTSS1, NAPRT1, NDUFB9, NOV, NRBP2, NSMCE2, OPLAH, PARP10, PHF20L1, PLEC, PPP1R16A, PTK2, PTP4A3, PUF60, PVT1, PYCRL, RECQL4, RHPN1, RHPN1.AS1, RNF139, SCRIB, SCRT1, SHARPIN, SLA, SLC39A4, SLC45A4, SLC52A2, SPATC1, SQLE, TIGD5, TMEM65, TMEM71, TNFRSF11B, TONSL, TOP1MT, TRAPPC9, TRIB1, TRMT12, TRPS1, TSTA3, VPS28, ZC3H3, ZFAT, ZFP41

***Functional enrichment of those genes (MSigDB C2 category, Bonferroni-corrected***
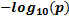
***):***

NIKOLSKY_BREAST_CANCER_8Q23_Q24_AMPLICON, 139.483967848423

AGUIRRE_PANCREATIC_CANCER_COPY_NUMBER_UP, 13.9610410158971

ONKEN_UVEAL_MELANOMA_UP, 5.5595399649524

DAVICIONI_MOLECULAR_ARMS_VS_ERMS_DN, 3.16089982649642

DEURIG_T_CELL_PROLYMPHOCYTIC_LEUKEMIA_UP, 2.94719563791031

PATIL_LIVER_CANCER, 2.31938878043002

**Immunoresponsive subtype:**

***Genes (association p value threshold is* 10^–5^*, gene count is 78):***

ABLIM1, ACTR1A, ADCY8, AFAP1L2, ASAP1, ASAP1.IT1, ATRNL1, BAG3, C10ORF118, C10ORF82, C10ORF95, C1ORF87, C8ORF31, CACUL1, CASC2, CCDC172, CUEDC2, CYP2J2, EFR3A, EIF3A, EMX2, EMX2OS, FAM135B, FAM160B1, FAM204A, FAM45B, FAM49B, FAM84B, FBXL15, GFRA1, GLI4, GPIHBP1, GRK5, GSDMC, HHLA1, HOOK1, HPYR1, HSPA12A, INA, INPP5F, KCNQ3, KIAA1598, LRRC6, LY6H, MCMBP, NANOS1, NFKB2, OMA1, PDZD8, PHF20L1, PNLIP, PNLIPRP1, PNLIPRP2, PNLIPRP3, PPAPDC1A, PRDX3, PRLHR, PSD, PTK2, PVT1, RAB11FIP2, RGS10, SEC23IP, SFXN2, SFXN4, SH3PXD2A, SLC18A2, SUFU, TACSTD2, TDRD1, TIAL1, TMEM180, TMEM71, TOP1MT, TRIB1, TRUB1, WDR11, ZFP41

***Functional enrichment of those genes (MSigDB C2 category, Bonferroni-corrected***
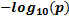
***):***

NIKOLSKY_BREAST_CANCER_8Q23_Q24_AMPLICON, 7.80574660503181

**Fibrous subtype:**

***Genes (association p value threshold is* 8 × 10^–4^*, gene count is 76):***

ABCA17P, ABCA3, AMDHD2, ATP6V0C, C16ORF59, CCDC114, CCDC64B, CCNF, CEMP1, CLDN6, CLDN9, COX6B2, EMP3, FAM71E2, FLYWCH1, FLYWCH2, FPR1, FPR2, FPR3, HAS1, HCFC1R1, IL11, IL32, ISOC2, KCTD5, KDELR1, MEFV, METTL22, MKLN1, MMP25, NAPSA, NAT14, NFKBIA, NLRP13, NLRP4, NLRP5, NLRP8, NTN3, OR1F1, OR1F2P, PDPK1, PODXL, PRSS21, PRSS22, PRSS27, PRSS30P, PRSS33, PSMA6, PTPRH, RPL28, SIGLEC5, SIGLEC6, SPACA6P, SRRM2, SRRM2.AS1, SSC5D, SUV420H2, SYNGR4, SYT5, TCEB2, THOC6, TMEM143, TMEM190, TMEM86B, TNFRSF12A, TOP1, ZG16B, ZNF114, ZNF175, ZNF200, ZNF205, ZNF205.AS1, ZNF213, ZNF350, ZNF480, ZNF528

***Functional enrichment of those genes (MSigDB C2 category, Bonferroni-corrected***
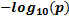
***):***

NIKOLSKY_BREAST_CANCER_16P13_AMPLICON, 20.8790876399383

**Supplementary Note 2: Subtype characterizations based on INSPIRE**

Specifications for the stromal subtype learned based on the INSPIRE latent variables was explained in the main text. Here we explain the other three INSPIRE subtypes: vascular, immunoresponsive, and fibrous.

The vascular subtype is characterized by low expression of modules 70, 71, 78, 79, and 81 (Fig. 5c), which indicates a strong association with the deactivation of immune system. Modules 70, 71, 78, and 79 are significantly enriched for the immune response, defense response and immune system process functions (Table S10). Module 81 is significantly enriched for caspase regulator activity, which modulates the activity of cysteine proteases involved in apoptosis (Table S10). Caspases are suggested to have many functions in the immune system besides acting as cell-death proteases [1]. The vascular subtype is also characterized by moderate or abundant vessel formation (Additional file: Figure S3B), which is potentially promoted by deactivation of immune system. Abundance of tumor vessels suggest fast tumor angiogenesis that facilitates the proliferation of tumor cells. The vascular subtype has a significant overlap (
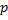
 = 6.81 × 10^–82^) with the proliferative subtype revealed by the TCGA consortium [2] (Table S11).

The immunoresponsive subtype is characterized by high expression of immune response, defense response, and immune system process modules 70, 71, and 78 (Table S10, Figure 5C) that are downregulated in vascular subtype. The vessel formation amount is minimal (Figure S7B-iv), which is potentially led by the high immune system response. The amount of stroma is low (Fig S3B-i) and the stroma is mostly fibrous, suggesting a lower tumor-stroma interaction than the stromal subtype, while both immunoresponsive and stromal subtypes show high immune system response. Immunoresponsive subtype has a significant overlap (
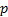
 = 6.54 × 10^–13^) with the immunoreactive subtype discovered by the TCGA study [2] (Table S11).

The fibrous subtype is characterized by high expression of modules 87 and 89 (Figure 5C), which are significantly enriched for G protein coupled receptor protein signaling pathway (Table S10), the series of molecular signals generated as a consequence of a G protein coupled receptor binding to its physiological ligand. The minimal desmoplasticity and minimal vessel formation in fibrous subtype (Fig S3B-ii,iv) suggest antiproliferative effects, which potentially depends, in part, on signal transduction via a G protein coupled receptor [3]. Fibrous subtype has a significant overlap (
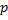
 = 2.17 × 10^–17^) with the differentiated subtype revealed by the TCGA consortium [2] (Table S11).

**REFERENCES:**

1. Hotchkiss RS, Nicholson DW. Apoptosis and caspases regulate death and inflammation in sepsis. Nat Rev Immunol. 2006;6:813–22.

2. Cancer Genome Atlas Research Network*.* Integrated genomic analyses of ovarian carcinoma. Nature 2011;474:609–15.

3. Yiu GK, Chan WY, Ng SW, Chan PS, Cheung KK, Berkowitz RS, et al. SPARC (secreted protein acidic and rich in cysteine) induces apoptosis in ovarian cancer cells. Am J Pathol. 2001;159:609–22.
