# Supplementary material for: Extracting a low-dimensional description of multiple gene expression datasets reveals a potential driver for tumor-associated stroma in ovarian cancer
Source: Genome Med. 2016 Jun 10;8:66. doi: 10.1186/s13073-016-0319-7 (PMC4902951; doi:10.1186/s13073-016-0319-7)
Supplement: Additional file 19: Table S11. — The confusion matrix representing the overlap between the INSPIRE subtypes and the subtypes revealed by the TCGA ovarian cancer study [23]. (DOC 99 kb) [file 13073_2016_319_MOESM19_ESM.doc]

**Table S11** The confusion matrix representing the overlap between the INSPIRE subtypes and the subtypes revealed by the TCGA ovarian cancer study^2^.

|  | | **TCGA subtypes** | | | |
| --- | --- | --- | --- | --- | --- |
|  |  | Proliferative | Mesenchymal | Immunoreactive | Differentiated |
| **INSPIRE subtypes** | Vas. | 103  (*p* = 6.81 × 10^–82^) | 8 | 2 | 10 |
|  | Str. | 0 | 82  (*p* = 1.03 × 10^–35^) | 12 | 10 |
|  | Imm. | 17 | 9 | 82  (*p* = 6.54 × 10^–13^) | 63 |
|  | Fib. | 16 | 10 | 9 | 52  (*p* = 2.17 × 10^–17^) |
