# Supplementary material for: Extracting a low-dimensional description of multiple gene expression datasets reveals a potential driver for tumor-associated stroma in ovarian cancer
Source: Genome Med. 2016 Jun 10;8:66. doi: 10.1186/s13073-016-0319-7 (PMC4902951; doi:10.1186/s13073-016-0319-7)
Supplement: Additional file 21: Table S12. — The six gene expression datasets we used in our pan-cancer survival analysis. (DOC 30 kb) [file 13073_2016_319_MOESM21_ESM.doc]

**Table S12** The six gene expression datasets we used in our pan-cancer survival analysis.

| **Name** | **Cancer type** | **Samples (n)** | **Genes (n)** | **Platform** |
| --- | --- | --- | --- | --- |
| Osloval | BRCA | 184 | 17,920 | Illumina HTv3 |
| GSE16011 | GBM | 159 | 18,113 | Affymetrix HGU133Plus2 + Agilent 244 k aCGH + Human Exon 1.0 |
| TCGA-GBM | GBM | 512 | 17,814 | Affymetrix HGU133A |
| Gentles | AML | 784 | 16,855 | Affymetrix HGU133Plus2 |
| GSE9899 | OV | 295 | 18,113 | Affymetrix HGU133Plus2 |
| TCGA-LUAD | LUAD | 395 | 16,256 | Illumina HiSeq |
